# Prognostic factors associated with outcomes after knee cartilage repair across different procedure types: a systematic review

**DOI:** 10.1186/s43019-026-00350-4

**Published:** 2026-10-20

**Authors:** Joo Hyung Han, Min Jung, Kwangho Chung, Hyun-Soo Moon, Se-Han Jung, Seung Hoon Baik, Jeongmo Koo, Sung-Hwan Kim

**Affiliations:** 1 Arthroscopy and Joint Research Institute, Yonsei University College of Medicine, Seoul, Republic of Korea; 2 Department of Orthopedic Surgery, Severance Hospital, Yonsei University College of Medicine, Seoul, Republic of Korea; 3 Department of Orthopedic Surgery, Yongin Severance Hospital, Yonsei University College of Medicine, Yongin-si, Republic of Korea; 4 Department of Orthopedic Surgery, Gangnam Severance Hospital, Yonsei University College of Medicine, Seoul, Republic of Korea

**Keywords:** Articular cartilage defect, Microfracture, Biologic augmentation, Prognostic factors, Systematic review

## Abstract

**Purpose:**

To identify and describe the prognostic factors associated with outcomes after knee cartilage repair within distinct procedure and disease contexts.

**Methods:**

PubMed, Embase, the Cochrane Library, and Google Scholar were searched from inception to 29 August 2026; backward and forward citation searching was also performed. Eligible reports quantitatively evaluated baseline factor–postoperative outcome associations after knee cartilage repair. Two reviewers independently selected reports, extracted association-level data, and applied the quality in prognosis studies tool. Because effect measures could not be harmonized, findings were synthesized without meta-analysis by disease context, procedure, factor, outcome, and follow-up.

**Results:**

Thirty-one reports were included, with report-level sample sizes totaling 13,022; cohort overlap prevented determination of the number of unique participants. Of 37 study–analysis units, 29 were at high risk-of-bias and 8 at moderate risk; none were at low risk. In isolated marrow stimulation, increasing age was associated with less favorable short- and long-term outcomes in two multivariable analyses at high overall risk-of-bias, whereas lesion-size findings varied across outcomes and follow-up. Autologous chondrocyte implantation (ACI)/matrix-induced autologous chondrocyte implantation (MACI) studies reported outcome-specific associations involving age, sex, body mass index, baseline status, defect characteristics, previous surgery, and manufacturing variables. Adjusted osteochondral-allograft failure analyses identified age, body mass index, diagnosis, graft area, and bony realignment as candidate factors; clinical-threshold and machine-learning findings remained context dependent. In osteoarthritis-associated cell-based cohorts, older age and bipolar lesions were associated with long-term failure in one adjusted analysis, while other findings were imprecise or derived from overlapping cohorts. No factor was consistently established across procedures or outcomes. A formal certainty-of-evidence framework was not applied because of marked heterogeneity, sparse context-specific evidence, and generally high risk-of-bias; definitive between-procedure comparisons were not possible.

**Conclusions:**

Available evidence identifies candidate prognostic associations within specific disease, procedure, outcome, and follow-up contexts. However, substantial clinical heterogeneity, potential confounding, cohort overlap, and sparse context-specific evidence limit the certainty and generalizability of the findings. The evidence does not support universal clinical cutoffs or conclusions that prognostic effects differ between procedures.

Level of evidence: level IV, systematic review of level I–IV studies.

**Supplementary Information:**

The online version contains supplementary material available at https://doi.org/10.1186/s43019-026-00350-4.

## Introduction

Focal cartilage defects of the knee are a frequent cause of pain and functional limitation; however, articular cartilage has a minimal intrinsic healing capacity [1]. Microfracture remains one of the most frequently performed cartilage repair techniques, while procedures incorporating biologic augmentation have been increasingly adopted to overcome the limitations of marrow stimulation [2, 3]. As these options continue to evolve, identifying the factors that influence clinical outcomes has become increasingly important.

Microfracture aims to restore joint surface integrity by penetrating the subchondral bone to release marrow-derived mesenchymal cells that form a fibrocartilaginous repair tissue [4]. Although widely accessible and cost-effective, its long-term durability is limited, particularly in larger lesions or high-demand patients [5]. Beyond isolated marrow stimulation, some cartilage-repair procedures combine marrow stimulation with a biologic adjunct [4, 6]. Cell-based cartilage implantation, including autologous or characterized chondrocyte implantation and certain mesenchymal stromal cell procedures, may be performed without marrow stimulation [7–10]. Osteochondral or scaffold-based procedures constitute another distinct therapeutic approach [4, 11]. These procedure categories differ substantially in biologic mechanism, surgical technique, and clinical indication, making their prognostic findings unsuitable for interpretation as a single augmented-procedure group [10, 12].

Several recent systematic reviews have provided valuable insights into prognostic factors after cartilage repair, including microfracture-specific evaluations and broader analyses across different joints or age groups [13–15]. However, procedure-specific prognostic evidence across mechanistically distinct cartilage-repair approaches has not been systematically mapped within separate disease contexts. A structured synthesis across distinct procedure and disease contexts may help identify potentially relevant prognostic factors while avoiding inappropriate comparisons between clinically heterogeneous populations.

Accordingly, the purpose of this systematic review was to identify and describe prognostic factors associated with outcomes after knee cartilage repair within distinct procedure categories and disease contexts. Prognostic findings were evaluated separately for focal cartilage defects and osteoarthritis-associated cartilage lesions.

## Methods

### Search strategy

The review protocol was registered in PROSPERO (CRD420251249852; https://www.crd.york.ac.uk/PROSPERO/view/CRD420251249852), and the review was reported in accordance with preferred reporting items for systematic reviews and meta-analyses [PRISMA] 2020. Several methodological elements, including procedure and disease-context stratification and prognosis-specific risk-of-bias assessment, were introduced after protocol registration to address heterogeneity in the included evidence. These deviations from the registered protocol are acknowledged as a limitation. These nonprespecified methods were applied to address the clinical and methodological heterogeneity of the included evidence. A structured literature search was conducted in PubMed, Embase, the Cochrane Library, and Google Scholar to identify studies examining prognostic factors for clinical outcomes after knee cartilage repair. Searches covered each source from inception and were updated on 29 August 2026. The final-scope strategy combined terms for knee cartilage conditions with procedure-specific concepts encompassing marrow stimulation, autologous or matrix-associated chondrocyte implantation, mesenchymal stromal or stem-cell procedures, stromal vascular fraction, bone marrow aspirate concentrate, osteochondral autograft or allograft transplantation, mosaicplasty, scaffold-based repair, and other cartilage-restoration procedures, together with prognosis and prediction concepts. No date or language filter was applied during retrieval; English-language eligibility was assessed during screening. Search syntax was adapted to each source. Complete source-specific search strategies, coverage, search dates, applied limits, and yields are provided in Supplementary Table 1. Five bounded Google Scholar queries were used, and the first 100 results from each query, ranked by relevance, were reviewed. Records identified across the sources were combined and duplicate entries were removed before title and abstract screening. Backward and forward citation searching of all included reports was also performed on 29 August 2026.

### Identification of eligibility

The inclusion criteria for this review were as follows: (1) studies involving adults aged 18 years or older undergoing knee cartilage repair for either a focal cartilage defect or an osteoarthritis-associated cartilage lesion; (2) studies evaluating isolated marrow stimulation, marrow stimulation combined with a biologic adjunct, cell-based cartilage implantation, or osteochondral/scaffold-based procedures; (3) studies that analyzed prognostic factors associated with clinical or radiological outcomes; (4) studies reporting quantitatively extractable associations between baseline characteristics and postoperative results; and (5) original peer-reviewed articles published in English. The exclusion criteria were as follows: (1) studies evaluating procedures outside the eligible knee cartilage-repair scope; (2) studies lacking an eligible prognostic-factor analysis or extractable postoperative association; (3) redundant reports from overlapping cohorts that contributed no unique eligible prognostic information; and (4) studies with ineligible designs, such as reviews, technical notes, case reports, editorials, or laboratory or animal investigations. Reports from overlapping cohorts were not automatically excluded. Multiple reports from the same or potentially overlapping cohort were retained when they provided unique eligible prognostic-factors, outcomes, or follow-up information. Overlapping reports retained in the review were linked and were not counted as independent evidence for the same factor–outcome–timepoint association. When a report met more than one exclusion criterion, one primary reason was assigned for PRISMA reporting. All records identified through the search were screened independently by two reviewers at the title and abstract levels, followed by a full-text assessment based on predefined criteria. Any discrepancies were resolved through discussion or, when necessary, consultation with a third reviewer.

### Procedure classification

Procedures were classified according to their primary therapeutic mechanism as follows: (1) isolated marrow-stimulation procedures; (2) marrow stimulation combined with a biologic adjunct; (3) cell-based cartilage implantation performed without marrow stimulation; and (4) osteochondral or scaffold-based procedures. Studies involving mixed treatment cohorts were identified separately. Study populations were classified into two disease contexts: (1) focal cartilage defects and (2) osteoarthritis-associated cartilage lesions. Classification was based on the eligibility criteria, diagnosis, radiographic osteoarthritis criteria, and lesion description reported in each primary study. Prognostic findings were synthesized separately for the two disease contexts.

### Data extraction and risk-of-bias assessment

Two reviewers independently extracted data from all studies that met the inclusion criteria. The extracted variables included the first author’s name, publication year, country, study design, level of evidence, sample size, disease context, lesion pathology, osteoarthritis diagnostic criteria, Kellgren–Lawrence grade, patient and lesion characteristics, procedure category, outcome measures, and reported prognostic factor–outcome associations. For each association, we also extracted the factor definition or cutoff, outcome and follow-up time point, statistical model, adjustment status, effect estimate with 95% confidence interval, and covariates, where reported. We additionally extracted study-level inclusion and exclusion criteria, recruitment period, lesion pathology, concomitant procedures, and follow-up completeness, as reported in the primary studies.

The review did not restrict eligibility to a closed prespecified list of prognostic factors. All baseline patient-, disease-, lesion-, and procedure-related variables evaluated as potential predictors in an eligible primary report were extracted irrespective of statistical significance. These included age, sex, body mass index (BMI), smoking, baseline patient-reported outcome or activity status, symptom duration, lesion size, lesion number and location, osteoarthritis severity or Kellgren–Lawrence grade, previous surgery, concomitant procedures, and treatment-related variables, where reported. Procedure-specific biologic variables, including cell-count or scaffold-related measures, were eligible only when defined at or before treatment and evaluated in relation to a postoperative outcome. Postoperative variables were not treated as baseline prognostic factors.

Potential cohort overlap was assessed across all included reports by comparing institution, recruitment period, eligibility criteria, treatment, and sample size. When reports were confirmed or considered likely to include overlapping participants, only one report was allowed to contribute to a given prognostic factor–outcome–time point synthesis. The report providing the most directly relevant and complete estimate, with preference for an adjusted analysis, was prioritized; overlapping reports were otherwise retained for descriptive reporting. The cohort-overlap assessment and handling decisions are presented in Supplementary Table 2.

Study-design-level methodological quality was assessed according to the original study design: randomized controlled trials were evaluated using the Cochrane risk-of-bias 2 (RoB 2) tool [16]. Nonrandomized cohort or case-series studies were assessed using the methodological index for nonrandomized studies (MINORS) [17]. Because these tools do not specifically assess bias in prognostic-factor associations, all included prognostic-factor analyses were assessed using the quality in prognosis studies (QUIPS) tool [18]. Two reviewers independently evaluated six domains: study participation, study attrition, prognostic-factor measurement, outcome measurement, study confounding, and statistical analysis and reporting. Associations derived from the same analysis or statistical model and sharing the same risk-of-bias profile were assigned a common QUIPS assessment ID. Each domain was judged as having low, moderate, or high risk-of-bias, and no numerical summary score was calculated. Disagreements were resolved through discussion until consensus was reached.

RoB 2 and MINORS served only as study-design-level descriptions and were not used to grade or interpret the prognostic-factor synthesis. The standard QUIPS signalling questions were applied at the analysis-unit level to consider source population selection, attrition and missingness, validity of prognostic-factor and outcome measurement, factor- and outcome-relevant confounding, and appropriateness and transparency of statistical analysis and reporting.

### Statistical analysis

A quantitative meta-analysis was not feasible because of the substantial heterogeneity across the studies in terms of design, patient populations, lesion characteristics, operative techniques, and outcome reporting. Mixed or comparative treatment studies contributed to a procedure-specific synthesis only when a prognostic association was estimated within the relevant treatment group or a factor-by-treatment interaction was evaluated. Associations estimated after pooling mechanistically distinct treatment groups were retained for descriptive reporting but were excluded from procedure-specific synthesis. Given these variations, we conducted a structured synthesis without meta-analysis. Findings were grouped hierarchically according to disease context, procedure category, prognostic factor, outcome domain, and follow-up horizon. Outcome domains were classified as final postoperative status, change from baseline, minimal clinically important difference (MCID) achievement, patient acceptable symptom state (PASS) achievement, failure or reoperation, activity, and structural imaging or arthroscopic outcomes. No single outcome domain was designated as primary because the objective was to map prognostic associations across clinically distinct outcome constructs. Each domain was therefore synthesized separately, and findings were not combined across final status, change, MCID, PASS, failure/reoperation, activity, or structural outcomes. Follow-up was categorized as short-term (≤ 2 years), mid-term (> 2 to < 5 years), or long-term (≥ 5 years). Original factor definitions, categories, and cutoffs were retained as reported in the primary studies; categorical or nonlinear associations were not reinterpreted as continuous linear effects.

Adjusted multivariable estimates were prioritized for interpretation. The adequacy of confounding control was considered in relation to the prognostic factor and outcome. Clinically relevant domains included age, sex, BMI, baseline outcome status, lesion size and location, previous surgery, osteoarthritis severity, and treatment group, where applicable; no model was assumed to be adequately adjusted solely because it was described as multivariable. Univariable comparisons, subgroup analyses, and correlations were reported separately and were not described as evidence of an independent prognostic association. All eligible findings were retained irrespective of statistical significance. For each association, interpretation considered the reported effect estimate and 95% confidence interval, direction and precision, adjustment status and covariates, QUIPS risk-of-bias, and cohort independence. A nonsignificant result was not interpreted as evidence of no association unless the reported confidence interval excluded a clinically important association. When multiple estimates were reported from the same cohort within the same factor–outcome–time stratum, all relevant estimates were displayed, but the cohort was counted only once. Results within a stratum were described as directionally consistent only when all independent cohort estimates with a reported direction pointed in the same direction; findings were described as mixed when opposite directions were reported and indeterminate when direction could not be established.

QUIPS judgments were incorporated qualitatively and were not converted into numerical weights. Overall risk-of-bias was classified as high when at least one QUIPS domain was high, moderate when at least one domain was moderate and no domain was high, and low only when all six domains were low. Because no analysis unit was judged to be at low overall risk-of-bias, a low-risk-only sensitivity analysis was not possible. A formal certainty-of-evidence framework was not applied because of the marked heterogeneity, sparse evidence within disease–procedure–outcome strata, and generally high risk-of-bias. Postoperative variables, including magnetic resonance imaging (MRI) defect fill measured after treatment, were not considered baseline prognostic factors; associations involving such variables were retained only as post-treatment correlations and excluded from the prognostic-factor synthesis. Because the number of studies within each disease–procedure–outcome stratum was small and no meta-analysis was performed, funnel plots and statistical tests for small-study effects were considered inappropriate. Risk-of-bias due to missing results was therefore not formally assessed. Potential publication bias and selective outcome reporting were considered qualitatively during interpretation. Results were presented using structured tables and visual displays generated in R (version 4.3.2; R Foundation for Statistical Computing, Vienna, Austria).

## Results

### Characteristics of included studies

**Fig. 1:**
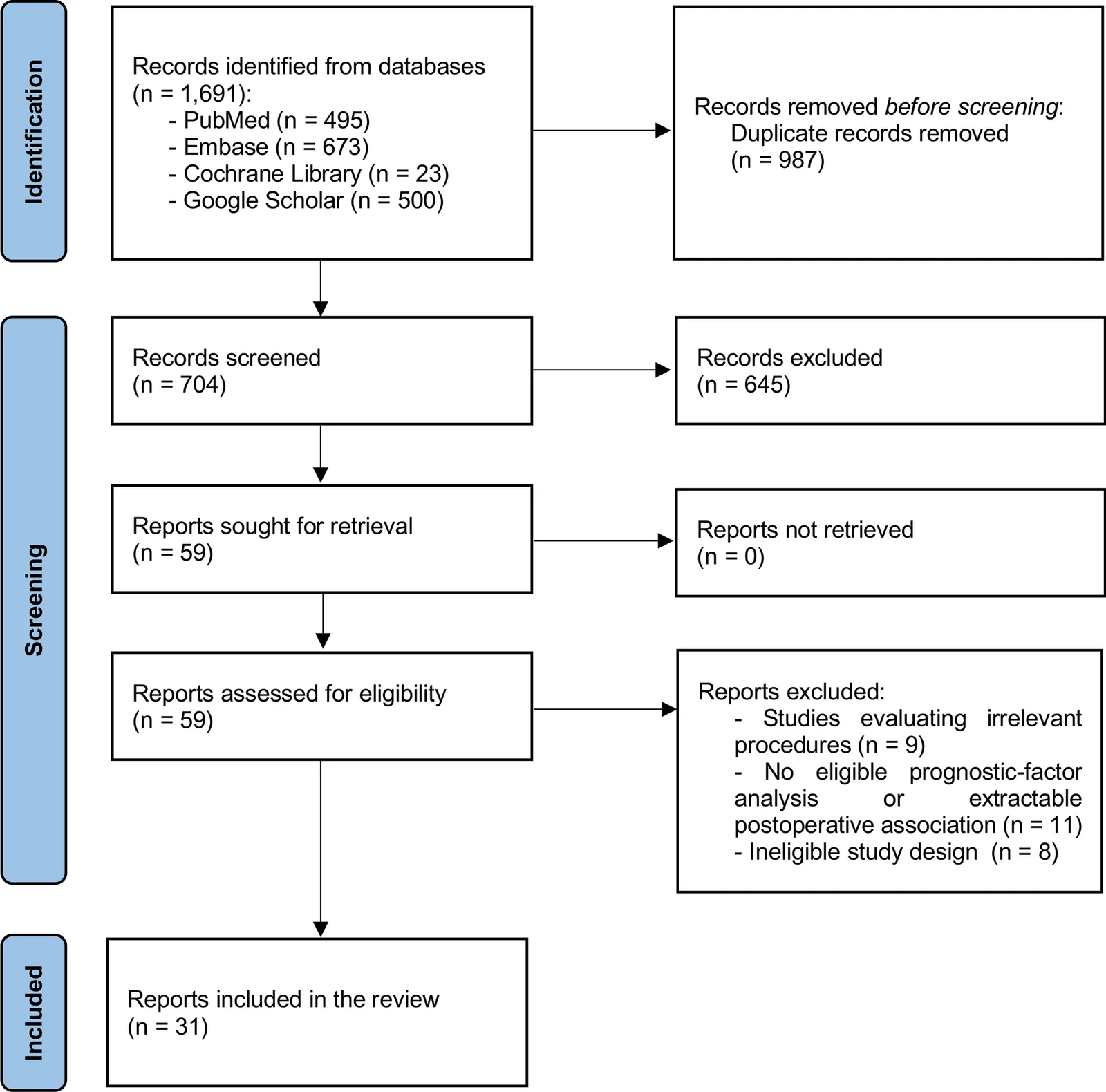
PRISMA flow diagram for the systematic review. PRISMA, preferred reporting items for systematic reviews and meta-analyses

A total of 1691 records were identified across the four search sources. After removal of 987 duplicate entries, 704 records were screened, of which 645 were excluded at the title and abstract stage. All 59 reports sought for retrieval were obtained and assessed for full-text eligibility. A total of 28 reports were excluded after full-text assessment, and 31 reports met the eligibility criteria and were included in the review (Fig. 1). Backward and forward citation searching identified no additional eligible reports. Report-level citations and primary reasons for the 28 full-text exclusions are provided in Supplementary Table 3. Reported sample sizes totaled 13,022; however, this is a sum across reports rather than a count of unique participants, and the number of unique participants could not be determined because of confirmed and possible cohort overlap. Of the 31 included reports, 26 evaluated focal cartilage defects, 4 evaluated osteoarthritis-associated cartilage lesions, and 1 included focal or mixed osteochondral diagnoses. One report provided level I evidence, 13 provided level III evidence, and 17 provided level IV evidence. The included procedures encompassed isolated marrow stimulation; marrow stimulation with or without a biologic adjunct; cell-based implantation using autologous chondrocytes, mesenchymal stromal cells, or related products; osteochondral autograft or allograft transplantation; scaffold-based procedures; and mixed or comparative cartilage-restoration cohorts. Most investigations were level III–IV observational studies [6–9, 11, 19–43], and one randomized controlled trial was included [44]. Detailed report characteristics are presented in Table 1; surgical techniques in Supplementary Table 4; and eligibility, recruitment, lesion, concomitant-procedure, and follow-up information in Supplementary Table 5.

**Table 1:** Characteristics of included reports

Authors	Year	Journal	Country	Study design	Level of evidence	Follow-up duration (mean)	Number of patients	Disease context	Procedure category	Main procedure	Outcome measures
Koch et al. [19]	2026	Knee Surg Sports Traumatol Arthrosc	Germany	Prospective case series	IV	11.3 years	112	Focal cartilage defect	Cell-based cartilage implantation without marrow stimulation	Matrix-associated autologous chondrocyte implantation	KOOS, IKDC, Lysholm, and EQ-5D
Philippe et al. [20]	2026	Life (Basel)	Switzerland	Prospective cohort study	IV	Mean 4.2 years (2-year and final assessments)	67 patients	Focal cartilage defect	Cell-based implantation without marrow stimulation	Second-generation autologous chondrocyte implantation (ACI)	KOOS, IKDC, and MOCART
Saad Berreta et al. [21]	2026	Orthop J Sports Med	USA	Retrospective cohort study	III	Minimum 5 years	70	Focal cartilage defect	Marrow stimulation with/without biologic adjunct (mixed cohort)	Primary arthroscopic microfracture; BMAC augmentation in a subset	IKDC and KOOS
Cook et al. [22]	2025	J Orthop	USA	Prospective registry cohort analyzed observationally	III	Mean 79 months; minimum 5 years for nonfailures	184 patients/186 knees	Focal cartilage defect	Osteochondral procedure	Osteochondral allograft transplantation (OCA/OCAT)	Treatment failure, revision OCA, and arthroplasty
Ebert et al. [23]	2025	Knee Surg Sports Traumatol Arthrosc	Australia	Retrospective analysis of a longitudinal MACI cohort	III	10 years	96 patients	Focal cartilage defect	Cell-based implantation without marrow stimulation	Matrix-induced autologous chondrocyte implantation (MACI)	10-year MRI composite score and graft tissue infill and KOOS Sport/Recreation
Tierney et al. [24]	2025	Knee Surg Sports Traumatol Arthrosc	UK	Prospective database analyzed retrospectively	IV	1–23 years; 1295 observations	306 patients	Focal cartilage defect	Cell-based implantation without marrow stimulation	Autologous chondrocyte implantation (ACI)	Longitudinal Lysholm score and joint failure
Wang et al. [25]	2025	Am J Sports Med	USA	Registry case series	IV	Mean 8.2 years; minimum 2 years	527 patients/560 knees	Focal/mixed osteochondral diagnoses	Osteochondral procedure	Osteochondral allograft transplantation (OCA)	Graft removal/conversion to arthroplasty and survivorship
Yazdi et al. [26]	2025	Am J Sports Med	USA	Retrospective cohort from a prospective database	III	Minimum 5 years	235 patients	Focal cartilage defect	Osteochondral procedure	Primary osteochondral allograft transplantation (OCA)	Lysholm, IKDC and KOOS MCID/PASS/SCB, and reoperation/failure
Sato et al. [27]	2024	Arthrosc Sports Med Rehabil	USA	Retrospective case series	IV	Mean 3.6 years; minimum 2 years	40 patients	Focal cartilage defect	Mixed cartilage-restoration cohort	Mosaicplasty, OCA, or MACI	Lysholm, PROMIS PF-CAT, VAS pain, and KOOS
Weishorn et al. [28]	2024	Am J Sports Med	Germany	Prospective case series with retrospective factor modeling	IV	Mean 8.1 years (range 5–11.9)	103 patients	Focal cartilage defect	Cell-based implantation without marrow stimulation	Matrix-induced autologous chondrocyte implantation (MACI)	KOOS, EQ-5D, VAS, Tegner, and MOCART 2.0
Bumberger et al. [29]	2023	Arthroscopy	Germany	Propensity-score-matched registry cohort	III	6–36 months	238 matched patients	Focal cartilage defect	Mixed cartilage-restoration cohort	ACI, BMS, osteochondral transfer and other restoration procedures	KOOS change, MCID, and revision/failure
Byrne et al. [30]	2023	Cartilage	USA	Retrospective claims-database cohort	IV	5 years	6391 patients	Focal cartilage defect	Osteochondral procedure	Osteochondral allograft transplantation (OCA)	Salvage cartilage procedure or arthroplasty
Kim et al. [6]	2023	J Exp Orthop	South Korea	Retrospective case series	IV	12 months	43	Osteoarthritis-associated cartilage lesion	Marrow stimulation combined with a biologic adjunct	Stromal vascular fraction implantation	VAS and MOCART
Gillinov et al. [31]	2022	Am J Sports Med	USA	Retrospective claims-database case series	IV	5 years	533 patients	Focal cartilage defect	Cell-based implantation without marrow stimulation	Autologous chondrocyte implantation (ACI)	Revision cartilage procedure or arthroplasty
Seiferth et al. [7]	2022	Am J Sports Med	Germany	Prospective cohort study	III	36 months	1494	Focal cartilage defect	Cell-based cartilage implantation without marrow stimulation	Autologous chondrocyte implantation	KOOS, VAS, patient satisfaction, and reintervention rate
Merkely et al. [32]	2021	Cartilage	USA	Retrospective cohort study	III	Mean time to failure 4.6 years (ACI) and 2.0 years (OCA)	368 patients (ACI 209; OCA 159)	Focal cartilage defect	Procedure-specific ACI and OCA cohorts	Autologous chondrocyte implantation or osteochondral allograft transplantation	Graft failure/revision/arthroplasty
Owusu-Akyaw et al. [33]	2021	Cartilage	USA	Retrospective cohort study	III	Mean 26.1 months	91 patients	Focal cartilage defect	Osteochondral procedure	Femoral osteochondral allograft transplantation (OCA)	2-year IKDC MCID achievement
Ramkumar et al. [34]	2021	Am J Sports Med	USA	Case–control machine-learning analysis	III	1 and 2 years	185 patients	Focal cartilage defect	Osteochondral procedure	Osteochondral allograft transplantation (OCA)	IKDC, KOS-ADL, and SF-36 MCID/SCB achievement
Ramkumar et al. [35]	2021	Am J Sports Med	USA	Case–control machine-learning analysis	III	2 years	185 patients (153 at 2 years)	Focal cartilage defect	Osteochondral procedure	Osteochondral allograft transplantation (OCA)	IKDC, KOS-ADL, and SF-36 MCID/SCB achievement
Wang et al. [11]	2021	Cartilage	USA	Retrospective cohort study	III	60 months	132	Focal cartilage defect	Osteochondral/scaffold procedure vs. isolated marrow stimulation	Biphasic synthetic scaffold vs. microfracture	SF-36, KOS-ADL, IKDC, Marx activity, and MRI integration/fill quality
Wright-Chisem et al. [36]	2021	Cartilage	USA	Retrospective cohort study	III	Minimum 2 years	245 patients	Focal cartilage defect	Osteochondral procedure	Osteochondral allograft transplantation (OCA)	MCID achievement for KOS-ADL, Marx, IKDC, and VAS outcomes
Chahla et al. [37]	2020	Am J Sports Med	USA	Retrospective case series	IV	24 months	206	Focal cartilage defect	Isolated marrow stimulation	Arthroscopic microfracture	IKDC and KOOS
Kim et al. [38]	2020	Orthop J Sports Med	South Korea	Retrospective case series	IV	Minimum 5 years; survival reported to 9 years	467 patients/483 knees	Osteoarthritis-associated cartilage lesion	Cell-based implantation without marrow stimulation	Adipose-derived MSC implantation on fibrin glue scaffold	IKDC, Tegner, and treatment survival/failure
Solheim et al. [39]	2018	J Orthop	Norway	Retrospective case series	IV	Median 14 years	205	Focal cartilage defect	Mixed marrow stimulation and osteochondral procedure	Arthroscopic microfracture or mosaicplasty	Lysholm score
Kim et al. [8]	2015	Am J Sports Med	South Korea	Retrospective cohort study	III	28.6 months	54	Osteoarthritis-associated cartilage lesion	Cell-based cartilage implantation without marrow stimulation	Arthroscopic mesenchymal stem cell implantation ± fibrin glue scaffold	IKDC, Tegner, and ICRS cartilage repair grade
Koh et al. [9]	2014	Am J Sports Med	South Korea	Retrospective case series	IV	26.5 months	35	Osteoarthritis-associated cartilage lesion	Cell-based cartilage implantation without marrow stimulation	Arthroscopic adipose-derived mesenchymal stem cell implantation	IKDC, Tegner activity scale, ICRS cartilage repair grade, and patient satisfaction
Vanlauwe et al. [44]	2011	Am J Sports Med	Belgium	Randomized controlled trial	I	60 months	112	Focal cartilage defect	Cell-based cartilage implantation vs. isolated marrow stimulation	Characterized chondrocyte implantation vs microfracture	KOOS, VAS, radiographic KL grade, and failure rate
Asik et al. [40]	2008	Arthroscopy	Turkey	Prospective case series	IV	68 months	90	Focal cartilage defect	Isolated marrow stimulation	Arthroscopic microfracture	Lysholm, Tegner, and Oxford knee score
Kreuz et al. [42]	2006	Arthroscopy	Germany	Prospective case series	IV	36 months	70	Focal cartilage defect	Isolated marrow stimulation	Arthroscopic microfracture	ICRS, modified Cincinnati score, and MRI (defect fill, cartilage signal, edema, effusion)
Kreuz et al. [41]	2006	Osteoarthritis cartilage	Germany	Prospective case series	IV	36 months	70	Focal cartilage defect	Isolated marrow stimulation	Arthroscopic microfracture	ICRS, modified Cincinnati score, and MRI (defect fill, cartilage signal, edema, effusion)
Steadman et al. [43]	2003	Arthroscopy	USA	Prospective case series	IV	11.3 years	68	Focal cartilage defect	Isolated marrow stimulation	Arthroscopic microfracture	Lysholm, Tegner, WOMAC, SF-36, and patient satisfaction

VAS, Visual analog scale; MOCART, Magnetic resonance observation of cartilage repair tissue; KOOS, Knee injury and osteoarthritis outcome score; KOS-ADL, Knee outcome survey–activities of daily living; IKDC, International knee documentation committee; SF-36, Short form-36 health survey; KL, Kellgren–Lawrence; MSC, Mesenchymal stem cell; ICRS, International cartilage repair society; USA, United States of America; WOMAC, Western Ontario and McMaster universities osteoarthritis index

Saad Berreta et al. evaluated a primary microfracture cohort in which a subset received bone marrow aspirate concentrate (BMAC) augmentation [21]. Cohort-wide prognostic models from this report were therefore classified as a mixed microfracture (MFx) ± BMAC augmentation context and were not attributed specifically to isolated MFx or BMAC augmentation; the unadjusted BMAC versus MFx subgroup comparison was reported separately. Mixed-treatment estimates from other reports were similarly assigned to a procedure-specific synthesis only when within-treatment estimates or factor–by–treatment interactions were available [11, 27, 29, 32, 39, 44].

The cohort-overlap assessment identified several confirmed or potentially overlapping report clusters. The two Kreuz reports described the same microfracture cohort [41, 42]. The cohort reported by Koh et al. was explicitly included within the nonscaffold group of Kim et al., and the later Kim et al. report was considered likely to represent an extended overlapping Yonsei cohort [8, 9, 38]. The two Ramkumar reports analyzed the same osteochondral allograft cohort [34, 35]. Additional possible or likely partial overlap was considered between the Rush microfracture reports [21, 37], among the German autologous chondrocyte implantation (ACI)/matrix-induced autologous chondrocyte implantation (MACI) reports [7, 19, 28, 29], and among the Hospital for Special Surgery osteochondral allograft reports [33–36]. Overlapping reports were retained when they provided unique factor, outcome, or follow-up information but were not counted as independent evidence for the same factor–outcome–time point unit. Detailed overlap evidence and handling decisions are provided in Supplementary Table 2.

### Methodological quality and risk-of-bias assessment

At the study-design level, 30 nonrandomized reports were assessed using MINORS. Four comparative reports had total scores of 19–20 [7, 8, 11, 29]. The remaining 26 noncomparative reports had a mean score of 11.5 ± 1.1 (range: 9–13). The single randomized controlled trial was assessed using RoB 2, with no domain judged to be at high risk-of-bias [44]. Detailed MINORS scores are presented in Supplementary Table 6, and the RoB 2 assessment is summarized in Supplementary Fig. 1.

The extraction comprised 98 association entries grouped into 37 study–analysis units. A total of 29 units were judged to be at high overall risk-of-bias and 8 at moderate risk; none were judged to be at low risk. High-risk judgments occurred predominantly in the study-attrition, study-confounding, and statistical-analysis/reporting domains. Post-treatment variables and machine-learning feature rankings that did not provide conventional adjusted effect estimates were identified separately and were not interpreted as equivalent to baseline independent prognostic associations [22, 28, 34, 35, 41, 42]. Association-level QUIPS IDs are provided in Supplementary Table 7, and the final consensus domain-level judgments and supporting rationales are presented in Supplementary Table 8.

### Structured synthesis of prognostic-factor associations

**Fig. 2:**
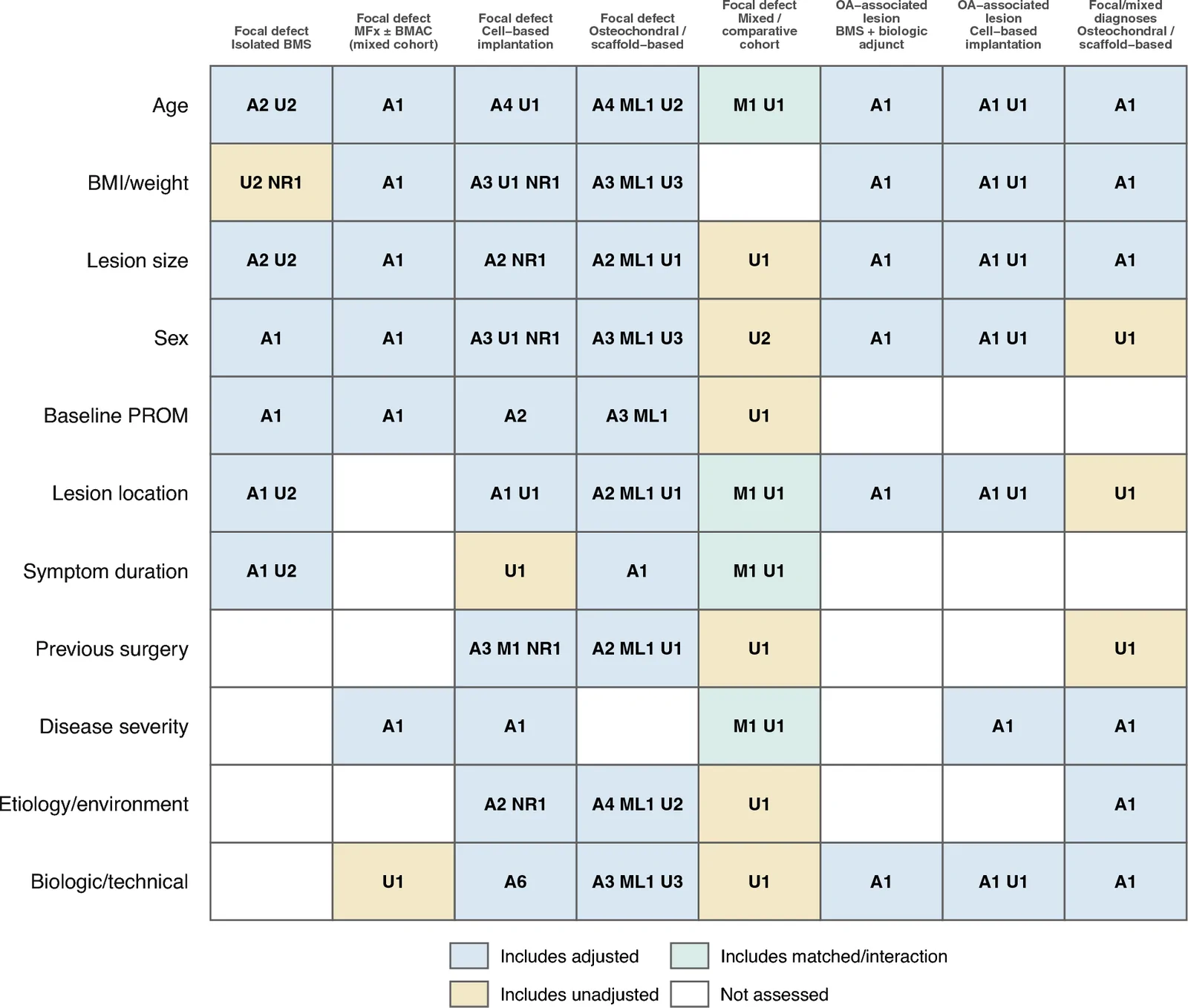
Context-specific coverage of prognostic-factor analyses. Cell labels report the number of coverage units contributing adjusted multivariable (A), matched or adjusted treatment-effect (M), machine-learning feature-ranking (ML), unadjusted (U), or incompletely reported factor-specific (NR) analyses within each disease and procedure context. Reports with confirmed cohort overlap were collapsed into one coverage unit when they contributed to the same factor and analysis category. Reports with possible cohort overlap were retained separately because participant-level linkage was unavailable; detailed overlap assessments are provided in Supplementary Table 2. Accordingly, coverage counts should not be interpreted as counts of fully independent patient cohorts when possible overlap remains unresolved. Tile colors distinguish analysis and reporting categories only and do not indicate association direction, magnitude, statistical significance, or certainty. The disease-context/procedure categories are: focal defect–isolated BMS; focal defect–MFx ± BMAC (mixed cohort); focal defect–cell-based implantation; focal defect–osteochondral/scaffold-based procedure; focal defect–mixed/comparative cohort; OA-associated lesion–BMS + biologic adjunct; OA-associated lesion–cell-based implantation; and focal/mixed osteochondral diagnoses–osteochondral/scaffold-based procedure. BMAC, bone marrow aspirate concentrate; BMI, body mass index; BMS, bone marrow stimulation; MFx, microfracture; OA, osteoarthritis; PROM, patient-reported outcome measure

**Fig. 3:**
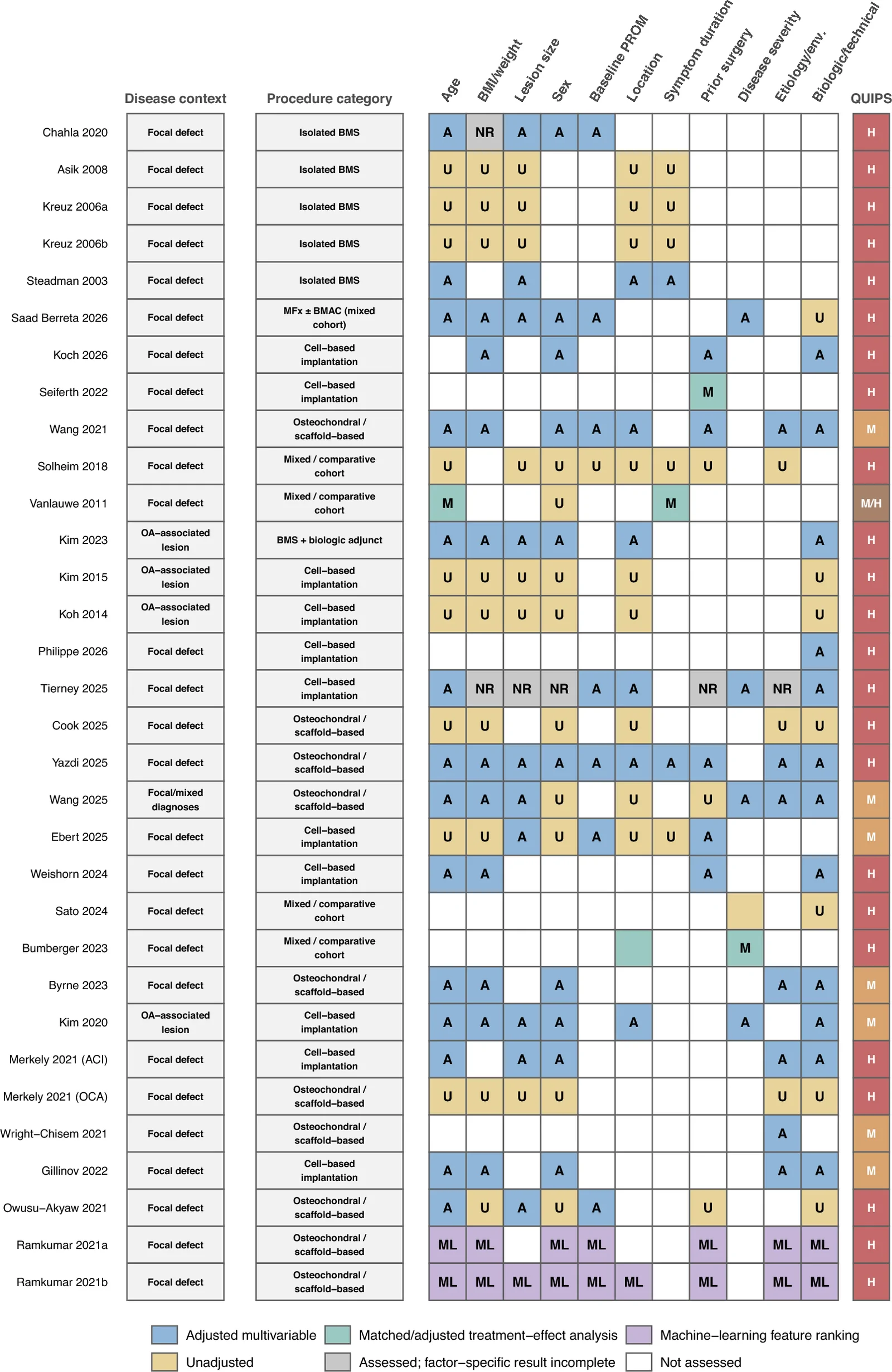
Study-level map of prognostic-factor assessment and methodological context. Evidence-map tiles indicate adjusted multivariable analysis (A), matched or adjusted treatment–effect analysis (M), machine-learning feature ranking (ML), unadjusted analysis (U), assessment with incomplete factor-specific reporting (NR), or no assessment. These categories do not indicate association direction, magnitude, statistical significance, or certainty. The QUIPS column shows the overall risk-of-bias as high (H), moderate (M), or varying from moderate to high across analysis units within the same study (M/H). The 31 included reports are displayed as 32 procedure-specific rows because Merkely et al. contributed separate ACI and OCA analyses. Disease context and procedure category are displayed in separate columns. The disease-context/procedure categories are: focal defect–isolated BMS; focal defect−MFx ± BMAC (mixed cohort); focal defect−cell-based implantation; focal defect−osteochondral/scaffold-based procedure; focal defect−mixed/comparative cohort; OA-associated lesion−BMS + biologic adjunct; OA-associated lesion−cell-based implantation; and focal/mixed osteochondral diagnoses−osteochondral/scaffold-based procedure. ACI, autologous chondrocyte implantation; BMAC, bone marrow aspirate concentrate; BMI, body mass index; BMS, bone marrow stimulation; MFx, microfracture; OA, osteoarthritis; OCA, osteochondral allograft; PROM, patient-reported outcome measure; QUIPS, quality in prognosis studies

Prognostic-factor associations were synthesized within separate disease, procedure, outcome, and follow-up contexts. Original factor coding and direction were retained, adjusted and unadjusted findings were distinguished, and overlapping cohorts were not counted as independent evidence. Detailed factor definitions, outcomes, follow-up time points, statistical models, effect estimates, confidence intervals, adjustment variables, and association-level QUIPS judgments are presented in Supplementary Table 7. Domain-level QUIPS judgments and supporting rationales are provided in Supplementary Table 8, and cohort-overlap assessments and handling decisions are reported in Supplementary Table 2. Because no analysis unit was judged to be at low overall risk-of-bias, no formal strong, moderate, or limited evidence grades were assigned. No formal assessment of reporting bias was conducted because the evidence was sparse, heterogeneous, and not meta-analyzed. Publication bias and selective outcome reporting therefore could not be excluded. Context-specific coverage of prognostic-factor analyses and the study-level map of assessed factors and methodological context are shown in Figs. 2 and 3, respectively.

Among focal-defect cohorts treated with isolated marrow stimulation, increasing age was associated with less favorable outcomes in two multivariable analyses at high overall risk-of-bias. Chahla et al. reported lower odds of MCID (OR, 0.61) and PASS (OR, 0.86) achievement at 24 months, although 95% confidence intervals were not reported, and Steadman et al. reported less long-term Lysholm improvement with increasing age (standardized B, −0.299; *P* = 0.011; 95% confidence interval not reported) [37, 43]. Unadjusted comparisons reported by Asik et al. and the overlapping Kreuz reports pointed in the same direction but were not treated as independent adjusted evidence [40–42]. Lesion-size findings were less uniform. Larger lesions were associated with lower 6-month MCID achievement in Chahla et al., whereas the adjusted long-term coefficient reported by Steadman et al. did not establish a clear association, and the favorable finding for defects < 2 cm^2^ versus ≥ 2 cm^2^ in Asik et al. was unadjusted [37, 40, 43].

Other findings after isolated marrow stimulation were specific to the outcome, time point, or factor coding. Male sex was associated with PASS achievement in the adjusted models of Chahla et al., whereas female patients had a higher unadjusted 5-year failure risk in the microfracture group of Vanlauwe et al. (RR, 4.78; 95% CI 1.49–15.62) [37, 44]. Higher baseline KOOS scores were associated with outcome-specific PASS achievement but were not generalized to MCID, change, or final-score outcomes. Lesion-location findings used anatomically different comparisons, and the available BMI and symptom-duration findings were predominantly unadjusted or incompletely reported [40–42].

In mixed or comparative focal-defect cohorts, the reported associations could not generally be attributed to an individual procedure. In the primary microfracture cohort reported by Saad Berreta et al., BMAC was administered to a subset; therefore, the cohort-wide associations involving age, sex, weight or BMI, lesion size, Kellgren–Lawrence grade, and baseline PROMs were retained as mixed MFx ± BMAC findings [21]. The unadjusted BMAC versus MFx comparison did not demonstrate a clear difference but involved only a small augmented subgroup. Sato et al. reported unadjusted correlations between greater preoperative whole-joint MRI degeneration and poorer selected postoperative PROMs across a mixed cartilage-restoration cohort [27]. In the German Cartilage Registry, bipolar lesions were associated with less KOOS improvement and lower 24-month MCID achievement than matched unipolar lesions, but the cohort included multiple cartilage-restoration procedures [29]. Within the scaffold group analyzed by Wang et al., increasing age and BMI and previous microfracture were associated with selected 2-year outcomes, although findings differed across outcomes and precision was incompletely reported [11]. The pooled microfracture–mosaicplasty findings of Solheim et al. were retained descriptively, and symptom duration in Vanlauwe et al. was treated as possible treatment–effect modification rather than as a procedure-specific prognostic association [39, 44].

Among focal-defect cohorts treated with ACI or MACI, associations varied according to the factor, outcome, and follow-up horizon. Koch et al. reported higher long-term PASS achievement among men and patients with a BMI of 20–29 kg/m^2^, but the analysis was at high risk-of-bias [19]. Tierney et al. reported lower 1-year Lysholm scores with increasing age and greater implanted-cell number, higher scores with a higher baseline Lysholm score and lateral rather than medial femoral condyle location, and a faster long-term decline for grade 4 than grade 3 defects [24]. In a moderate-risk 10-year structural analysis, larger defect size was associated with a lower MRI composite score and less tissue infill, and more previous knee procedures were associated with less tissue infill [23]. Weishorn et al. reported lower long-term KOOS scores with increasing BMI and more previous knee surgeries; the association with follow-up MOCART was considered a post-treatment correlation rather than a baseline prognostic factor [28].

Procedure-specific biologic and failure analyses provided additional but nonuniform findings. Philippe et al. reported greater improvement in selected PROMs with a higher initial chondrocyte yield and less improvement with a longer preimplantation population-doubling time, but 95% confidence intervals and complete covariate information were not reported [20]. Previous cartilage treatment was associated with less favorable subsequent KOOS scores in the registry analysis of Seiferth et al., although a common effect estimate was not reported [7]. In the ACI cohort of Merkely et al., larger defect size and hypertension were associated with failure; the published female-sex coefficient and odds ratio were internally inconsistent and were not reinterpreted [32]. In a claims-based ACI cohort, female sex and BMI ≥ 35 kg/m^2^ were associated with higher adjusted odds of revision within 5 years [31]. Differences in factor definitions, outcomes, and potential cohort overlap prevented these findings from being interpreted as independent replications of common associations (Table 2).

**Table 2:** Study-level map of prognostic-factor evidence and its role in the structured synthesis

Disease and procedure context	Study	Factor profile and observed direction	Outcome domain and follow-up horizon	Analysis status and overall QUIPS	Cohort independence and role in synthesis
Focal cartilage defect Mixed microfracture ± BMAC cohort	Saad Berreta et al. [21]	Weight (continuous): increasing weight → lower IKDC MCID/PASS achievement. Baseline PROM (continuous): lower score → higher MCID achievement. Lesion size (continuous): increasing size → lower IKDC/KOOS Sport MCID achievement. BMI (continuous): increasing BMI → lower MCID/PASS achievement. KL grade 0–1 versus 2–3: higher PASS achievement. Age (continuous): increasing age → lower PASS achievement. Male versus female: higher KOOS Symptoms MCID achievement. BMAC augmentation versus MFx alone: no clear difference; estimate/CI NR	MCID and PASS; mean 7.1 years (long-term)	AIC-selected multivariable models (Q01; high risk). BMAC versus MFx subgroup comparison unadjusted (Q02; high risk)	Possible partial overlap with Chahla et al. [37] could not be excluded. Pooled mixed-treatment associations retained descriptively and not attributed specifically to MFx or BMAC augmentation
Focal cartilage defect Isolated marrow stimulation	Chahla et al. [37]	Lesion size (continuous and > 2 cm^2^): increasing size → lower MCID achievement at 6 months. Age (reported as continuous): increasing age → lower MCID/PASS achievement at 24 months. Male versus female: higher PASS achievement at 6 and 24 months. Baseline KOOS Symptoms/Pain (continuous): higher score → higher outcome-specific PASS achievement. At 12 months, factor-specific estimates were NR	MCID and PASS; 6, 12, and 24 months (short-term)	Multivariable logistic regression (Q07; high risk)	Possible partial overlap with Saad Berreta et al. [21] could not be excluded. Retained in the isolated-MFx synthesis but not treated as independent replication for a shared stratum
	Asik et al. [40]	Age < 35 versus ≥ 35 years, defect < 2 cm^2^ versus ≥ 2 cm^2^, nonweightbearing versus weightbearing location, and BMI < 25 versus ≥ 25 kg/m^2^: the first category was associated with better final scores. Symptoms < 12 versus ≥ 12 months: estimate/direction NR	Final Lysholm, Tegner, and Oxford scores; mean 68 months (long-term)	Unadjusted subgroup/Spearman analyses (Q12; high risk)	No overlap identified; counted as one independent cohort
	Kreuz et al. [41]	Femoral-condyle versus other locations: better clinical/structural outcomes. Age < 40 versus ≥ 40 years: better ICRS outcome in the younger group. Lesion size, BMI, defect grade, and symptom duration: estimate/direction NR. Postoperative MRI fill correlated with ICRS but was not treated as a baseline prognostic factor	Final ICRS/Cincinnati and structural outcomes; 36 months (mid-term)	Unadjusted group/correlation analyses (Q13a/Q13b; high risk)	Confirmed same cohort as Kreuz et al. [42]. Used for lesion-location reporting; duplicate factor–outcome–time estimates counted once. Post-treatment MRI correlation excluded from prognosis synthesis
	Kreuz et al. [42]	Age ≤ 40 versus > 40 years: better ICRS/Cincinnati scores in the younger group. Femoral condyle versus tibial/patellofemoral locations: better outcomes. Lesion size, BMI, and symptom duration: estimate/direction NR. Postoperative MRI fill correlated with ICRS but was not treated as a baseline prognostic factor	ICRS/Cincinnati and structural outcomes; 6–36 months (short- to mid-term)	Unadjusted subgroup/correlation analyses (Q14a/Q14b; high risk)	Confirmed same cohort as Kreuz et al. [41]. Used for the more detailed age analysis; not counted as an independent cohort. Post-treatment MRI correlation excluded from prognosis synthesis
	Steadman et al. [43]	Age (continuous): increasing age → less Lysholm improvement. Chronicity, lesion location, and lesion size: negative coefficients, but the reported estimates did not establish clear independent directions; 95% CIs NR	Change in Lysholm score; mean 11.3 years (long-term)	Backward multivariable linear regression (Q15; high risk)	No overlap identified; counted as one independent cohort
Focal cartilage defect Cell-based implantation without marrow stimulation	Koch et al. [19] (MACI)	Male versus female and BMI 20–29 kg/m^2^ versus other BMI categories: higher PASS achievement. Immature versus mature articular cartilage and > 1 versus ≤ 1 previous knee surgery: estimates were imprecise	PASS across PROMs; ≥ 10 years (long-term)	Multivariable logistic regression (Q03; high risk)	Possible overlap with the German registry cohort in Seiferth et al. [7] could not be excluded. Retained in the long-term PASS stratum and not treated as independent registry replication
	Philippe et al. [20]	Initial chondrocyte yield > 10^5^: greater 4-year improvement in KOOS Symptoms, Pain, ADL and Sport. Longer preimplantation population-doubling time: less improvement in IKDC, KOOS Pain and ADL. ACAN/COL2A1 expression and final confluence: no clear associations	Change in PROMs and MOCART; mean 4.2 years (mid-term)	Multiple linear regression; 95% CIs NR (Q16; high risk)	Independent cohort; manufacturing-factor evidence retained at the reported mid-term horizon
	Ebert et al. [23]	Larger defect size: lower 10-year MRI composite score and lower tissue infill. More prior knee procedures: lower tissue infill. Other tested baseline demographic/clinical variables were not clearly associated	10-year structural MRI outcomes (long-term)	Multivariable linear/ordinal logistic models (Q21; moderate risk)	Independent long-term structural-outcome cohort
	Tierney et al. [24]	Each additional year of age and greater log implanted-cell number: lower 1-year Lysholm. Higher baseline Lysholm and lateral femoral condyle versus medial location: higher 1-year Lysholm. Grade 4 versus grade 3 defect: faster long-term decline	Longitudinal Lysholm; 1–23 years	Multilevel longitudinal model (Q17; high risk)	Independent longitudinal ACI cohort; short-term level and long-term slope estimates kept separate
	Weishorn et al. [28]	Higher BMI and more previous knee surgeries: lower long-term KOOS; higher MOCART 2.0: higher KOOS. Age and implanted-cell count were not clearly associated in the multivariable model	Final KOOS; mean 8.1 years (long-term)	Multivariable linear regression, *n* = 59 (Q22; high risk)	Retained for long-term KOOS/MOCART estimates; not counted as independent replication of Koch for a shared factor–outcome–time unit
	Gillinov et al. [31]	Female versus male sex and BMI ≥ 35 versus normal BMI: higher adjusted odds of revision within 5 years. Age, comorbidity score, and bony realignment were not clearly associated	Revision surgery; 5 years (long-term)	Multivariable claims-based logistic regression (Q29; moderate risk)	Retained as a separate ACI claims cohort
	Seiferth et al. [7] (ACI)	Previous knee surgery versus none and previous cartilage treatment at the index defect versus none: lower subsequent KOOS/satisfaction in matched comparisons. Previous BMS versus previous ACI: no clear difference; common effect estimate/CI NR	KOOS, VAS, reoperation, and satisfaction; 6–36 months (short- to mid-term)	Propensity-score-matched comparisons (Q05a/Q05b; high risk)	Possible overlap with Koch et al. [19] could not be excluded. Reported separately by outcome/time and not interpreted as independent replication for a shared stratum
OA-associated cartilage lesion Cell-based implantation without marrow stimulation	Kim et al. [38]	Age ≥ 70 versus < 50 years and bipolar kissing lesions: higher hazard of MSC-implantation failure. Overall implanted-cell-number term was significant, but individual category CIs were imprecise; sex, BMI, side, lesion location, and lesion size were not clearly associated	Treatment survival/failure; 5–9 years (long-term)	Multivariable Cox regression (Q26; moderate risk)	Retained for unique long-term Cox survival estimates; not treated as independent replication of earlier short-/mid-term Yonsei findings
	Kim et al. [8] (MSC ± fibrin glue)	BMI ≥ 27.5 versus < 27.5 kg/m^2^ and lesion size ≥ 5.7 versus < 5.7 cm^2^: poorer IKDC/Tegner outcomes. Lesion size was associated with arthroscopic ICRS repair grade. Age, sex, lesion location, and subchondral cyst: estimate/direction NR	Final IKDC/Tegner and arthroscopic ICRS grade; mean 28.6 months (mid-term)	Unadjusted subgroup/correlation analyses (Q09; high risk)	Confirmed overlap with Koh et al. [9] for 35 patients/37 knees in the nonscaffold group. Shared factor–outcome–time estimates counted once; scaffold-specific results retained separately
	Koh et al. [9] (AD-MSC local adherent)	BMI ≥ 27.5 versus < 27.5 kg/m^2^ and lesion size ≥ 5.4 versus < 5.4 cm^2^: poorer IKDC, Tegner, and ICRS outcomes. Age, sex, lesion location, and subchondral cyst: estimate/direction NR	Final IKDC/Tegner and arthroscopic ICRS grade; mean 26.5 months (mid-term)	Unadjusted subgroup analyses (Q10; high risk)	Confirmed overlap with the nonscaffold group of Kim et al. [8]; not counted as independent evidence for the same factor–outcome–time point
OA-associated cartilage lesion Marrow stimulation plus biologic adjunct	Kim et al. [6] (SVF + drilling)	Lesion size (< 3.5 [reference], 3.5–5.5, 5.5–7.5, and ≥ 7.5 cm^2^): larger categories → higher odds of an unsatisfactory outcome. Lower SVF cell-count categories: higher odds of an unsatisfactory outcome. Age, sex, side, BMI, and lesion location: sparse/imprecise estimates	Composite unsatisfactory VAS/MOCART outcome; 12 months (short-term)	Multivariable logistic regression (Q04; high risk)	No overlap identified with the earlier Yonsei cohorts; counted as one independent cohort
Focal cartilage defect Mixed/comparative cartilage-restoration cohort	Sato et al. [27]	Greater whole-joint MRI degeneration (maximum WORMS), and meniscal/cartilage abnormalities, correlated with poorer selected postoperative PROMs; bone-marrow-edema-like lesion score correlated with worse VAS pain. Mixed procedure estimates were not procedure-specific	Final PROMs; mean 3.6 years (mid-term)	Unadjusted Spearman correlations (Q23; high risk)	Independent mixed-procedure evidence; correlations retained descriptively and not assigned to an individual procedure
	Bumberger et al. [29]	Bipolar versus unipolar lesion status: smaller KOOS improvement at 6, 12, 24, and 36 months and lower 24-month MCID achievement after propensity matching. Revision failure rate and time to failure did not differ clearly	KOOS change/MCID and failure; ≤ 36 months	Propensity-score-matched comparisons (Q24; high risk)	Retained only for the unique propensity-matched bipolar versus unipolar analysis; shared registry factors do not constitute independent replication
	Merkely et al. [32]	ACI: larger defect size and hypertension predicted higher failure; female-sex estimate was reported as significant but the published coefficient and OR are internally inconsistent. ACI age and smoking were not independently associated. OCA: no candidate factor entered a multivariable model	Graft failure; procedure-specific follow-up	ACI multivariable logistic regression; OCA unadjusted (Q27; high risk)	Independent report. ACI multivariable estimates retained; OCA findings remained unadjusted because no factor entered a multivariable model
	Solheim et al. [39] (microfracture and mosaicplasty; pooled analysis)	Single lesion, normal surrounding cartilage, baseline Lysholm ≥ 50, symptoms < 36 months, no patellofemoral involvement, age ≤ 25 years, and defect ≤ 3 cm^2^: better long-term outcome in unadjusted analyses. Reverse categories and partial meniscectomy were reported for poor outcome. Treatment, sex, trauma, side, and meniscal factors: estimate/direction NR	Good/poor Lysholm classification; median 14 years (long-term)	Unadjusted dichotomized analyses (Q08; high risk)	No overlap identified. MFx and mosaicplasty were pooled without within-treatment estimates or interaction testing; retained descriptively and excluded from procedure-specific synthesis
	Vanlauwe et al. [44] (CCI versus microfracture)	Symptom duration < 3 versus ≥ 3 years: possible modification of the CCI–MFx treatment effect; the exact interaction estimate was NR. Female versus male: higher unadjusted failure risk within both CCI and MFx groups. Age < 35 versus ≥ 35 years: no procedure-specific prognostic estimate	Change in overall KOOS and failure/reintervention; 60 months (long-term)	Adjusted treatment-effect analysis (Q11a; moderate risk); sex-specific and age analyses Q11b/Q11c (high risk)	No overlap identified. Within-group estimates and factor-by-treatment evidence retained; pooled treatment contrasts were not reclassified as prognostic factors
Focal cartilage defect Osteochondral/scaffold-based procedure	Cook et al. [22]	Concurrent ligament reconstruction, osteotomy and tibiofemoral bipolar-plus-other-surface OCA were associated with failure in unadjusted analyses; older age and higher BMI differed between failure and survival groups. Postoperative nonadherence was excluded as a baseline factor	Treatment failure; mean 79 months (long-term)	Unadjusted group comparisons/reported ORs (Q18; high risk)	Independent report. Baseline patient/procedure factors retained; postoperative rehabilitation adherence excluded from the baseline prognostic synthesis
	Yazdi et al. [26]	Male sex: lower odds of IKDC MCID and SCB. More previous surgery, workers’ compensation, longer symptom duration and condylar/trochlear location predicted lower achievement for selected PROM thresholds; major concomitant procedures predicted higher achievement for selected outcomes. Lower baseline PROM often predicted change-threshold achievement	MCID/PASS/SCB; ≥ 5 years (long-term)	Outcome-specific multivariable logistic models (Q19; high risk)	Kept separate from Rush MFx cohorts; multivariable threshold-outcome estimates retained
	Byrne et al. [30]	Age 20–29 versus 50–59 years and concomitant/prior bony realignment: lower adjusted odds of salvage surgery. Sex, BMI, comorbidity score, tobacco, diabetes, hypertension and vitamin-D deficiency were not clearly associated	Salvage surgery; 5 years (long-term)	Multivariable claims-based logistic regression (Q25; moderate risk)	Retained as a separate OCA claims cohort; cross-procedure concordance is not treated as replication
	Owusu-Akyaw et al. [33]	Lower baseline SF-36 PCS and defect diameter > 2 cm: higher odds of achieving the 2-year IKDC MCID. Age and baseline MCS estimates were imprecise	IKDC MCID; 2 years (short-term)	Multivariable logistic regression (Q30; high risk)	Preferred conventional adjusted OR/CI source for baseline PCS/MCS and defect size versus 2-year IKDC MCID
	Ramkumar et al. [34]	Lower baseline mental health and activity demand, worse pain symptoms, and higher baseline physical/knee function or BMI were prominent features of failure to achieve selected MCID/SCB outcomes. Feature importance is not an adjusted effect estimate	MCID/SCB; 1 and 2 years (short-term)	Machine-learning models with SHAP; no factor-specific CI (Q31; high risk)	Retained for unique mental-health, pain-catastrophizing and activity-demand features; shared PROM/demographic associations counted once
	Ramkumar et al. [35]	Higher BMI, malalignment, absence of preoperative osseous edema, concomitant ACL/meniscal injury, larger defect size and > 1 OCA graft were prominent features of failure to achieve 2-year functional MCID/SCB. Feature importance is not an adjusted effect estimate	MCID/SCB; 2 years (short-term)	Machine-learning models with SHAP; no factor-specific CI (Q32; high risk)	Retained for unique imaging/malalignment features; shared PROM/demographic associations counted once
	Wang et al. [11] (multivariable models restricted to the synthetic scaffold group)	Age and BMI (continuous): increasing values → poorer outcome for selected PROM/activity models. Previous microfracture versus none: poorer IKDC. Traumatic versus nontraumatic etiology and male versus female: directions differed by outcome. Higher baseline score → higher corresponding final score. Lesion location and scaffold-plug number: imprecise estimates	Final SF-36 PCS, KOS-ADL, IKDC, and Marx activity; 2 years (short-term)	Multivariable linear regression (Q06; moderate risk)	No overlap identified. Prognostic models were restricted to the 66-patient scaffold group; no MFx-specific prognostic association was inferred
	Wright-Chisem et al. [36]	Number of self-reported drug allergies was not clearly associated with MCID achievement for IKDC, Marx, VAS pain or health state; KOS-ADL showed an imprecise trend	MCID achievement; ≥ 2 years (short-term)	Logistic regression adjusted for age, BMI and sex (Q28; moderate risk)	Retained for the unique drug-allergy analysis only
Focal/mixed osteochondral diagnoses Osteochondral/scaffold-based procedure	Wang et al. [25]	Age ≥ 30 years, BMI ≥ 30, degenerative chondral lesion, avascular necrosis, osteoarthritis and total graft area > 8 cm^2^: higher adjusted odds of OCA failure. Location, previous surgery and multiple grafts were univariate-only findings	Graft failure/survival; mean 8.2 years (long-term)	Multivariable logistic regression (Q20; moderate risk)	Independent OCA registry; multivariable failure estimates prioritized

Exact factor coding, outcome definitions, follow-up time points, statistical models, effect estimates with 95% CIs, and adjustment covariates are provided in Supplementary Table 7. Cohort-overlap assessments and domain-level QUIPS judgments with supporting rationales are provided in Supplementary Tables 2 and 8, respectively. Findings described as unclear or NR should not be interpreted as evidence of no association. Because some reports may represent overlapping cohorts, report-level sample sizes should not be interpreted as counts of unique participants. Short-term was defined as ≤ 2 years, mid-term as > 2 to < 5 years, and long-term as ≥ 5 years

ACAN, Aggrecan; ACI, autologous chondrocyte implantation; ACL, anterior cruciate ligament; AD-MSC, adipose-derived mesenchymal stem cell; AIC, Akaike information criterion; BMAC, bone marrow aspirate concentrate; BMI, body mass index; BMS, bone marrow stimulation; CCI, characterized chondrocyte implantation; CI, confidence interval; COL2A1, Collagen type II alpha 1 chain; ICRS, International cartilage repair society; IKDC, International knee documentation committee; KL, Kellgren–Lawrence; KOOS, Knee injury and osteoarthritis outcome score; KOS-ADL, Knee outcome survey–activities of daily living; M-ACI, matrix-associated autologous chondrocyte implantation; MCID, Minimal clinically important difference; MCS, Mental component summary; MFx, Microfracture; MOCART, magnetic resonance observation of cartilage repair tissue; MRI, magnetic resonance imaging; MSC, mesenchymal stem cell; NR, not reported; OA, osteoarthritis; OCA, osteochondral allograft; OR, odds ratio; PASS, patient acceptable symptom state; PCS, Physical component summary; PROM, Patient-reported outcome measure; QUIPS, Quality in prognosis studies; SCB, substantial clinical benefit; SF-36, 36-item short form health survey; SHAP, SHapley Additive exPlanations; SVF, stromal vascular fraction; VAS, visual analog scale; WORMS, whole-organ magnetic resonance imaging score

In osteochondral allograft cohorts, adjusted long-term failure analyses identified different patient, diagnosis, and graft factors. Wang et al. reported higher adjusted odds of failure for age ≥ 30 years, BMI ≥ 30 kg/m^2^, degenerative chondral lesions, avascular necrosis, osteoarthritis, and total graft area > 8 cm^2^.[25] Byrne et al. reported lower adjusted odds of salvage surgery among patients aged 20–29 versus 50–59 years and among those undergoing prior or concomitant bony realignment [30]. In the outcome-threshold models of Yazdi et al., male sex, previous surgery, workers’ compensation, symptom duration, lesion location, and concomitant procedures showed outcome-specific associations, but the directions could not be generalized across MCID, PASS, and substantial clinical benefit [26]. Cook et al. reported unadjusted associations of concurrent ligament reconstruction, osteotomy, and extensive bipolar grafting with failure; postoperative treatment adherence was excluded from the baseline prognostic synthesis [22].

Short-term OCA analyses were similarly context dependent. Lower baseline SF-36 physical scores and defect diameter > 2 cm were associated with greater odds of achieving the 2-year IKDC MCID, which represents a change-threshold outcome rather than a more favorable final score [33]. The number of self-reported drug allergies was not clearly associated with most MCID outcomes after adjustment for age, BMI, and sex [36]. Two reports from the same OCA cohort used machine-learning feature rankings to identify mental health, baseline function, activity, pain, BMI, malalignment, osseous edema, associated injury, defect size, and graft number as prominent model features; these rankings were not interpreted as conventional adjusted effect estimates and were counted once for overlapping outcomes [34, 35]. In the OCA subgroup reported by Merkely et al., no candidate factor was retained in a multivariable failure model [32].

In osteoarthritis-associated cell-based cohorts, Kim et al. reported that age ≥ 70 versus < 50 years and the presence of bipolar kissing lesions were associated with higher hazards of long-term MSC-implantation failure [38]. The overall implanted-cell-number term was statistically significant, but the confidence intervals for the individual cell-number categories crossed 1. In a smaller SVF cohort, larger lesion-size and lower cell-count categories were associated with increasing odds of an unsatisfactory 12-month outcome, but the estimates were highly imprecise and the analysis was at high risk-of-bias [6]. Earlier reports described unadjusted BMI and lesion-size threshold associations, but the cohorts overlapped and were not counted as independent evidence [8, 9]. Across all disease and procedure contexts, heterogeneous factor coding, outcome domains, follow-up horizons, incomplete reporting of precision, potential cohort overlap, sparse context-specific evidence, and generally high risk-of-bias limited confidence in the findings and did not support definitive comparisons between procedures.

## Discussion

The principal finding of this review was that prognostic associations were highly dependent on procedure, disease context, factor coding, outcome construct, and follow-up horizon. Increasing age showed a similar unfavorable direction in two adjusted isolated-marrow-stimulation analyses, although both were at high risk-of-bias and precision was incompletely reported [37, 43]. The expanded ACI/MACI and OCA evidence identified additional candidate patient, lesion, manufacturing, and operative factors, but no factor could be regarded as consistently established across procedures or outcomes [23, 25]. The marked heterogeneity, sparse context-specific evidence, and generally high risk-of-bias limited the certainty of the findings. Accordingly, a formal certainty-of-evidence framework was not applied, and the evidence did not support conclusions that prognostic effects differed between procedures.

The present review differs methodologically from previous reviews rather than merely compiling a larger list of statistically significant factors. Van Tuijn et al. [13] focused specifically on clinical outcomes after knee microfracture and reported that older age, larger lesion size, longer symptom duration, and previous ipsilateral knee surgery were frequently associated with less favorable outcomes, although effect-size reporting was incomplete and quantitative pooling was not possible. Migliorini et al. [14] evaluated a broader population that included knee and ankle lesions treated using multiple surgical techniques, limiting direct comparison with procedure- and disease-specific knee cohorts. The incremental contribution of the present review is the association-level extraction of factor coding, outcome and follow-up context, adjustment status, effect estimates and precision, prognosis-specific QUIPS assessment, and explicit evaluation of cohort overlap. These methods showed that several apparently repeated findings arose from overlapping cohorts, mixed-treatment analyses, machine-learning feature rankings, or mathematically different outcome constructs; therefore, these findings should not be interpreted as independent confirmation.

Within isolated marrow stimulation, increasing age remained a potentially relevant candidate prognostic factor. Chahla et al. reported lower short-term MCID and PASS achievement with increasing age, and Steadman et al. reported less long-term Lysholm improvement in older patients [37, 43]. Unadjusted findings from Asik et al. and the overlapping Kreuz reports pointed in the same direction but did not establish independent associations [40–42]. Lesion-size findings require greater caution. Larger lesions were associated with lower short-term MCID achievement in Chahla et al., whereas the adjusted long-term estimate in Steadman et al. did not establish a clear association, and the favorable finding for defects smaller than 2 cm^2^ in Asik et al. was unadjusted. Findings for sex, baseline PROMs, lesion location, BMI, and symptom duration also differed according to the outcome definition and factor coding [37, 44]. Accordingly, the evidence does not support a universal age, lesion-size, or other baseline cutoffs for isolated marrow stimulation.

The expanded cell-based implantation literature likewise did not identify a single reproducible prognostic profile. Reported associations involved sex and BMI categories, age, baseline function, defect grade and location, lesion size, previous surgery, previous cartilage treatment, hypertension, and manufacturing variables [7, 19, 20, 23, 24, 28, 31, 32]. However, these associations related to different outcomes, including PASS, longitudinal PROM scores, structural MRI findings, graft failure, and revision. The direction of an association also depended on its coding. For example, a BMI of 20–29 kg/m^2^ was associated with greater PASS achievement in one category-based analysis, whereas increasing BMI or BMI ≥ 35 kg/m^2^ was associated with lower long-term KOOS or higher revision risk in other analyses [19, 28, 31]. Similarly, implanted-cell number, initial chondrocyte yield, and population-doubling time represented different biologic constructs and should not be interpreted as mutually confirmatory findings [20, 24]. Potential overlap among registry and institutional cohorts further limited interpretation as independent replication.

The osteochondral allograft literature added several adjusted failure and outcome-threshold models but remained context dependent. Older age, higher BMI, degenerative or osteoarthritic diagnoses, avascular necrosis, and larger total graft area were associated with failure in one long-term registry model, whereas younger age and bony realignment were associated with a lower risk of salvage surgery in a claims-based analysis [25, 30]. These results should not be combined directly with MCID, PASS, or substantial-clinical-benefit models. In particular, lower baseline physical function and a larger defect were associated with greater odds of achieving an MCID in one study, which reflects the opportunity for postoperative change rather than a more favorable final clinical state [33]. Other outcome-specific associations involved sex, previous surgery, workers’ compensation, symptom duration, lesion location, concomitant procedures, and extensive bipolar grafting [22, 26]. Machine-learning feature rankings provided potentially useful predictive information but did not supply conventional adjusted factor-specific estimates, and the two machine-learning reports represented the same cohort [34, 35]. Possible overlap among additional Hospital for Special Surgery reports also precluded treating these findings as fully independent replications [33, 36].

The osteoarthritis-associated cell-based evidence remained distinct from the focal-defect literature. Age ≥ 70 years and bipolar kissing lesions were associated with higher hazards of long-term MSC-implantation failure in the expanded Yonsei cohort, whereas the individual implanted-cell-number category estimates were imprecise [38]. A smaller SVF study reported lesion-size and cell-count category associations with an unsatisfactory composite outcome, but the confidence intervals were very wide [6]. Earlier reports described unadjusted BMI and lesion-size threshold associations, but the cohorts overlapped and were not counted as independent evidence [8, 9]. These findings may inform hypotheses for osteoarthritis-associated cartilage lesions but should not be generalized to focal cartilage defects or interpreted as replicated evidence.

Interpretation was also constrained by differences in treatment indication and perioperative context. Procedure selection was influenced by disease context, lesion characteristics, previous treatment, and patient characteristics, creating indication bias that could not be separated from treatment-specific prognostic effects. Rehabilitation protocols and return-to-activity criteria varied or were incompletely reported and may have affected time-dependent patient-reported and activity outcomes. Concomitant procedures, where present, could modify joint loading and clinical outcomes, thereby limiting attribution of an observed association to the cartilage procedure alone. Lesion chronicity was inconsistently represented by symptom duration or previous surgery and was insufficiently standardized for cross-study comparison. Structural outcomes also differed in imaging modality, assessment criteria, and follow-up timing and were therefore interpreted separately from clinical outcomes.

Biologic explanations for the observed associations remain plausible but should be separated from the review findings. Age-related changes in cellular activity and the greater mechanical demands imposed by larger lesions could potentially contribute to less favorable cartilage repair [4, 15]. Likewise, mechanical loading and metabolic factors associated with BMI may affect cartilage-repair outcomes [45, 46]. However, the included studies did not directly test these mechanisms, and no reliable factor-by-treatment evidence demonstrated that biologic augmentation compensated for age-related decline or altered the effect of BMI. These mechanisms therefore remain explanatory hypotheses rather than conclusions of the present review.

Clinically, the reported factors should be considered candidate contextual variables rather than validated selection criteria. The available evidence does not support excluding patients or selecting a cartilage-repair procedure based on a single age, BMI, lesion-size, symptom-duration, cell-count, or imaging threshold. Prognostic estimates should be interpreted according to whether the outcome represents final postoperative status, change from baseline, achievement of a clinical threshold, structural repair, or treatment failure. Future studies should prespecify prognostic models, report adjusted estimates with confidence intervals, use harmonized outcome domains and follow-up horizons, and evaluate factor–by–treatment interactions when treatment-specific differences are proposed. Participant-level linkage or transparent reporting of registry and institutional cohorts will also be necessary to prevent overlapping populations from being interpreted as independent replication.

### Limitations

This systematic review has several limitations. First, the evidence base consisted predominantly of observational prognostic analyses. Of the 37 study–analysis units assessed using QUIPS, 29 were judged to be at high overall risk-of-bias and 8 at moderate risk; none was judged to be at low risk. Residual confounding, incomplete follow-up, data-dependent model selection, multiple outcome-specific models, and inconsistent multivariable adjustment may therefore have influenced the reported associations, which should not be interpreted as causal effects. Second, substantial heterogeneity in disease context, procedure, factor coding, outcome definition, and follow-up time point precluded meta-analysis and estimation of pooled prognostic effects. Effect estimates, confidence intervals, and adjustment covariates were also frequently incompletely reported, particularly for nonsignificant findings. Consequently, a lack of statistical significance could not generally be interpreted as evidence of no clinically important association. Machine-learning feature rankings also could not be interpreted as equivalent to conventional adjusted effect estimates. Third, confirmed and possible cohort overlap affected several report clusters, including the Kreuz microfracture reports, the Yonsei MSC reports, the Ramkumar OCA reports, and additional Rush, German ACI/MACI, and Hospital for Special Surgery cohorts [7–9, 19, 21, 28, 29, 33–38, 41, 42]. Although overlapping estimates were not counted as independent evidence for the same factor–outcome–time point unit, participant-level identifiers were unavailable, and the sum of report-level sample sizes overestimates the number of unique participants. Fourth, several procedure and outcome contexts remained represented by single studies or mixed-treatment cohorts, and differences between separate literatures could not establish factor–by–treatment effects. Finally, the final-scope search was expanded after the review scope had been broadened, and the registered protocol did not prespecify the procedure taxonomy, disease-context and outcome/time-horizon stratification, association-level extraction framework, structured synthesis, QUIPS assessment, or cohort-overlap audit. In addition, because the final-scope search incorporated prognosis and prediction concepts, secondary prognostic analyses that did not use related terminology in the title or abstract may have been missed. Google Scholar screening was bound to the first 100 relevance-ranked results for each of five queries, and the search strategy did not undergo formal peer review of electronic search strategies (PRESS)-style review by an information specialist. These search limitations, post hoc methodological amendments, restriction to English-language publications, incomplete primary-study reporting, and potential publication or selective outcome reporting bias provide additional sources of uncertainty.

## Conclusions

Available evidence identifies candidate prognostic associations within specific disease, procedure, outcome, and follow-up contexts. However, substantial clinical heterogeneity, sparse context-specific evidence, generally high risk-of-bias, potential confounding, and cohort overlap limit the certainty and generalizability of the findings. The evidence does not support universal clinical cutoffs or conclusions that prognostic effects differ between procedures.

## Supplementary Information

Additional file 1.

## Data Availability

No datasets were generated or analyzed during the current study.

## Abbreviations

ACI: Autologous chondrocyte implantation
ACL: Anterior cruciate ligament
AD-MSC: Adipose-derived mesenchymal stem cell
ADL: Activities of daily living
AIC: Akaike information criterion
BMAC: Bone marrow aspirate concentrate
BMI: Body mass index
BMS: Bone marrow stimulation
CCI: Characterized chondrocyte implantation
CI: Confidence interval
EQ-5D: EuroQol 5-dimension
ICRS: International cartilage repair society
IKDC: International knee documentation committee
KL: Kellgren–Lawrence
KOOS: Knee injury and osteoarthritis outcome score
KOS-ADL: Knee outcome survey–activities of daily living
M-ACI: Matrix-associated autologous chondrocyte implantation
MACI: Matrix-induced autologous chondrocyte implantation
MCID: Minimal clinically important difference
MCS: Mental component summary
MFx: Microfracture
MINORS: Methodological index for nonrandomized studies
MOCART: Magnetic resonance observation of cartilage repair tissue
MRI: Magnetic resonance imaging
MSC: Mesenchymal stem cell
NR: Not reported
OA: Osteoarthritis
OCA: Osteochondral allograft
OR: Odds ratio
PASS: Patient acceptable symptom state
PCS: Physical component summary
PRISMA: Preferred reporting items for systematic reviews and meta-analyses
PROM: Patient-reported outcome measure
PROMIS PF-CAT: Patient-reported outcomes measurement information system physical function computer adaptive test
PROSPERO: International prospective register of systematic reviews
QUIPS: Quality in prognosis studies
RoB 2: Version 2 of the Cochrane risk-of-bias tool for randomized trials
RR: Risk ratio
SCB: Substantial clinical benefit
SF-36: 36-Item short form health survey
SHAP: SHapley additive exPlanations
SVF: Stromal vascular fraction
USA: United States of America
VAS: Visual analog scale
WOMAC: Western Ontario and McMaster universities osteoarthritis index
WORMS: Whole-organ magnetic resonance imaging score
