# Supplementary material for: Prognostic factors associated with outcomes after knee cartilage repair across different procedure types: a systematic review
Source: Knee Surg Relat Res. 2026 Oct 20;38:49. doi: 10.1186/s43019-026-00350-4 (PMC13682157; doi:10.1186/s43019-026-00350-4)
Supplement: Supplementary file 1 [file 43019_2026_350_MOESM1_ESM.docx]

**Supplementary Figure 1.** Risk of bias assessment of the included randomized study using the RoB 2.0 tool. (a) Summary plot showing the proportion of judgments across the five RoB 2.0 domains. (b) Traffic‐light plot illustrating domain-level and overall risk‐of-bias judgments for the study.

**Supplementary Table 1.** Full database-specific search strategies and source-level yields.

| Database/platform | Full search strategy | Search date | Records retrieved or screened |
| --- | --- | --- | --- |
| PubMed (National Library of Medicine) | (("Knee Joint"[Mesh] OR knee[tiab] OR knees[tiab]) AND ("Cartilage, Articular"[Mesh] OR cartilage[tiab] OR chondral[tiab] OR osteochondral[tiab]) AND (microfracture[tiab]  OR "micro-fracture"[tiab]  OR microdrilling[tiab]  OR "micro-drilling"[tiab]  OR "subchondral drilling"[tiab]  OR "bone marrow stimulation"[tiab]  OR "marrow stimulation"[tiab]  OR nanofracture[tiab]  OR "autologous matrix-induced chondrogenesis"[tiab]  OR AMIC[tiab]  OR "autologous chondrocyte implantation"[tiab]  OR "autologous chondrocyte transplantation"[tiab]  OR "matrix-associated chondrocyte implantation"[tiab]  OR "matrix-induced chondrocyte implantation"[tiab]  OR "characterized chondrocyte implantation"[tiab]  OR MACI[tiab]  OR "mesenchymal stem cell*"[tiab]  OR "mesenchymal stromal cell*"[tiab]  OR "adipose-derived stem cell*"[tiab]  OR "adipose-derived stromal cell*"[tiab]  OR "stromal vascular fraction"[tiab]  OR SVF[tiab]  OR "bone marrow aspirate concentrate"[tiab]  OR BMAC[tiab]  OR "osteochondral autograft*"[tiab]  OR "osteochondral allograft*"[tiab]  OR "osteochondral transplant*"[tiab]  OR "osteochondral autograft transfer"[tiab]  OR "osteochondral plug*"[tiab]  OR mosaicplast*[tiab]  OR scaffold*[tiab]  OR "cartilage repair"[tiab]  OR "cartilage restoration"[tiab]  OR "cartilage transplant*"[tiab]  OR "cartilage implant*"[tiab]  OR "minced cartilage"[tiab]  OR "particulated juvenile cartilage"[tiab])) AND (prognos*[tiab]  OR predict*[tiab]  OR predictor*[tiab]  OR "risk factor*"[tiab]  OR determinant*[tiab]  OR "factors associated"[tiab]  OR "factor associated"[tiab]  OR "independent factor*"[tiab]  OR "influencing factor*"[tiab]) | August 29, 2026 | 495 |
| Embase.com (Elsevier) | ('knee'/exp OR knee:ti,ab,kw OR knees:ti,ab,kw) AND ('articular cartilage'/exp OR cartilage:ti,ab,kw OR chondral:ti,ab,kw OR osteochondral:ti,ab,kw) AND (microfracture:ti,ab,kw  OR 'micro-fracture':ti,ab,kw  OR microdrilling:ti,ab,kw  OR 'micro-drilling':ti,ab,kw  OR 'subchondral drilling':ti,ab,kw  OR 'bone marrow stimulation':ti,ab,kw  OR 'marrow stimulation':ti,ab,kw  OR nanofracture:ti,ab,kw  OR 'autologous matrix-induced chondrogenesis':ti,ab,kw  OR amic:ti,ab,kw  OR 'autologous chondrocyte implantation':ti,ab,kw  OR 'autologous chondrocyte transplantation':ti,ab,kw  OR 'matrix-associated chondrocyte implantation':ti,ab,kw  OR 'matrix-induced chondrocyte implantation':ti,ab,kw  OR 'characterized chondrocyte implantation':ti,ab,kw  OR maci:ti,ab,kw  OR 'mesenchymal stem cell*':ti,ab,kw  OR 'mesenchymal stromal cell*':ti,ab,kw  OR 'adipose-derived stem cell*':ti,ab,kw  OR 'adipose-derived stromal cell*':ti,ab,kw  OR 'stromal vascular fraction':ti,ab,kw  OR svf:ti,ab,kw  OR 'bone marrow aspirate concentrate':ti,ab,kw  OR bmac:ti,ab,kw  OR 'osteochondral autograft*':ti,ab,kw  OR 'osteochondral allograft*':ti,ab,kw  OR 'osteochondral transplant*':ti,ab,kw  OR 'osteochondral autograft transfer':ti,ab,kw  OR 'osteochondral plug*':ti,ab,kw  OR mosaicplast*:ti,ab,kw  OR scaffold*:ti,ab,kw  OR 'cartilage repair':ti,ab,kw  OR 'cartilage restoration':ti,ab,kw  OR 'cartilage transplant*':ti,ab,kw  OR 'cartilage implant*':ti,ab,kw  OR 'minced cartilage':ti,ab,kw  OR 'particulated juvenile cartilage':ti,ab,kw) AND (prognos*:ti,ab,kw  OR predict*:ti,ab,kw  OR predictor*:ti,ab,kw  OR 'risk factor*':ti,ab,kw  OR determinant*:ti,ab,kw  OR 'factors associated':ti,ab,kw  OR 'factor associated':ti,ab,kw  OR 'independent factor*':ti,ab,kw  OR 'influencing factor*':ti,ab,kw) | August 29, 2026 | 673 |
| Google Scholar | Query 1 knee (microfracture OR microdrilling OR "bone marrow stimulation" OR nanofracture OR AMIC) (prognosis OR predictor OR "risk factor" OR "factors associated")  Query 2 knee ("autologous chondrocyte implantation" OR "matrix-induced chondrocyte implantation" OR "matrix-associated chondrocyte implantation" OR MACI OR "characterized chondrocyte implantation") (prognosis OR predictor OR "risk factor" OR "factors associated")  Query 3 knee ("osteochondral allograft" OR "osteochondral autograft" OR OATS OR mosaicplasty OR "osteochondral transplant") (prognosis OR predictor OR "risk factor" OR "factors associated")  Query 4 knee ("mesenchymal stem cell" OR "mesenchymal stromal cell" OR "stromal vascular fraction" OR SVF OR BMAC) (cartilage OR chondral OR osteochondral) (prognosis OR predictor OR "risk factor" OR "factors associated")  Query 5 knee ("cartilage repair" OR "cartilage restoration" OR scaffold OR "minced cartilage" OR "particulated juvenile cartilage") (prognosis OR predictor OR "risk factor" OR "factors associated") | August 29, 2026 | 500 |
| Cochrane Library (Wiley) | #1 MeSH descriptor: [Knee Joint] explode all trees #2 (knee OR knees):ti,ab,kw #3 #1 OR #2 #4 MeSH descriptor: [Cartilage, Articular] explode all trees #5 (cartilage OR chondral OR osteochondral):ti,ab,kw #6 #4 OR #5 #7 (microfracture OR "micro-fracture" OR microdrilling OR "micro-drilling" OR "subchondral drilling" OR "bone marrow stimulation" OR "marrow stimulation" OR nanofracture OR "autologous matrix-induced chondrogenesis" OR AMIC OR "autologous chondrocyte implantation" OR "autologous chondrocyte transplantation" OR "matrix-associated chondrocyte implantation" OR "matrix-induced chondrocyte implantation" OR "characterized chondrocyte implantation" OR MACI OR "mesenchymal stem cell*" OR "mesenchymal stromal cell*" OR "adipose-derived stem cell*" OR "adipose-derived stromal cell*" OR "stromal vascular fraction" OR SVF OR "bone marrow aspirate concentrate" OR BMAC OR "osteochondral autograft*" OR "osteochondral allograft*" OR "osteochondral transplant*" OR "osteochondral autograft transfer" OR "osteochondral plug*" OR mosaicplast* OR scaffold* OR "cartilage repair" OR "cartilage restoration" OR "cartilage transplant*" OR "cartilage implant*" OR "minced cartilage" OR "particulated juvenile cartilage"):ti,ab,kw #8 (prognos* OR predict* OR predictor* OR "risk factor*" OR determinant* OR "factors associated" OR "factor associated" OR "independent factor*" OR "influencing factor*"):ti,ab,kw #9 #3 AND #6 AND #7 AND #8 | August 29, 2026 | 23 |
| Coverage was from database inception. PubMed, Embase.com, the Cochrane Library, and Google Scholar were redesigned and rerun on August 29, 2026. The Cochrane Library search yielded 23 records, and the five bounded Google Scholar searches yielded 500 result entries. These entries did not add any unique eligible reports beyond those identified through the other sources. Google Scholar screening was limited to the first 100 relevance-ranked results for each query. Backward and forward citation searching of all 31 included reports was also completed on August 29, 2026, with no additional studies identified. No date or language filter was applied during database retrieval. | | | |

**Supplementary Table 2.** Assessment of potential cohort overlap among the included reports.

| Report | Institution/setting | Recruitment period | Eligibility/population | Procedure | Sample size | Overlap assessment | Handling in synthesis |
| --- | --- | --- | --- | --- | --- | --- | --- |
| Saad Berreta et al., 2026 | Rush University Medical Center, Chicago, USA; single-institution prospective database | Jan 2013–Sep 2018 | Symptomatic tibiofemoral cartilage defect after failed nonoperative care; primary MFx cohort with BMAC augmentation in a subset; minimum 5-year follow-up | Primary arthroscopic MFx; BMAC augmentation in a subset | 70 patients | Possible partial overlap with Chahla 2020: same Rush database, overlapping operative period (2013–Jan 2017), and shared surgeons/authors; patient identifiers were unavailable. | Cohort-wide prognostic models were treated as a mixed MFx ± BMAC augmentation context and were not attributed specifically to isolated MFx or BMAC augmentation. The unadjusted BMAC-versus-MFx subgroup comparison was reported separately. Not treated as independent replication of Chahla 2020. |
| Chahla et al., 2020 | Rush University Medical Center, Chicago, USA; institutional cartilage preservation repository | Jan 2004–Jan 2017 | Symptomatic knee pain after failed nonoperative care; primary MFx; minimum 2-year follow-up | Primary arthroscopic MFx | 206 patients | Possible partial overlap with Saad Berreta 2026 because of the shared database and overlapping operative period; patient identifiers were unavailable. | Synthesized only at 6-, 12-, and 24-month time points. Not treated as independent replication of Saad Berreta across time horizons. |
| Solheim et al., 2018 | Deaconess University Hospital/Aleris Nesttun Hospital, Bergen, Norway; local prospective registry | 1998–2003 | Age ≤60 years; symptomatic focal full-thickness chondral lesion; no marked joint-space loss, malalignment, or ligament instability | MFx or mosaicplasty | 205 patients (121 MFx; 84 mosaicplasty) | No overlap identified with another included report. Earlier follow-up reports cited by the authors were not included in this review. | Pooled mixed-treatment prognostic estimates retained descriptively and excluded from procedure-specific synthesis. |
| Seiferth et al., 2022 | German Cartilage Registry (KnorpelRegister DGOU); nationwide multicenter registry (>100 centers) | Registry initiated 2013; data through Dec 2020 | Adults undergoing knee cartilage surgery; ACI recipients with intact menisci selected for the reported matched analyses | ACI | 1,494 patients eligible; matched analyses: 730 vs 690 and 317 vs 254 | Overlap with Koch 2026 cannot be excluded because the registry is multicenter and overlaps calendar time; site-level participant identifiers were unavailable. | Reported separately by outcome and follow-up; not interpreted as independent replication of Koch for a shared factor–outcome–time stratum. |
| Wang et al., 2021 | Hospital for Special Surgery, New York, USA; prospective institutional registry | Registry screening 1999–2014 | Skeletally mature patients with one symptomatic grade III/IV femoral or trochlear lesion (1–6 cm²) and minimum 2-year follow-up | Biphasic synthetic scaffold or MFx | 132 patients (66 per group) | No overlap identified with another included report. | Prognostic models were restricted to the 66-patient scaffold group; no MFx-specific prognostic estimate was inferred. |
| Kim et al., 2023 | Yonsei Sarang Hospital, Seoul, Korea | Sep 2019–Apr 2021 | Symptomatic knee OA unresponsive to nonoperative care; 12-month clinical and MRI follow-up | SVF implantation with fibrin glue after subchondral drilling | 43 patients | No overlap with the included 2010–2012 Yonsei cohorts based on the nonoverlapping treatment period. | Counted as an independent cohort. |
| Kim et al., 2015 | Yonsei Sarang Hospital, Seoul, Korea | Oct 2010–Apr 2012 | Isolated full-thickness cartilage lesion in KL grade 1–2 OA; second-look arthroscopy after MSC implantation | MSC implantation with or without fibrin-glue scaffold | 54 patients/56 knees (37/39 without scaffold; 17/17 with scaffold) | Confirmed overlap with Koh 2014: 35 patients/37 knees in the non-scaffold group were explicitly identified as included in the prior report. | For the shared local-adherent MSC cohort, each factor–outcome–time estimate was counted once. Scaffold-specific results were retained separately. |
| Koh et al., 2014 | Yonsei Sarang Hospital, Seoul, Korea | Oct 2010–Sep 2011 | Isolated full-thickness cartilage lesion in KL grade 1–2 OA; no previous surgery; voluntary second-look arthroscopy | Local-adherent adipose-derived MSC implantation without scaffold | 35 patients/37 knees in the prognostic analysis | Confirmed overlap with the non-scaffold group of Kim 2015 (35 patients/37 knees). | Not counted as independent from Kim 2015 for the same factor–outcome–time point. |
| Vanlauwe et al., 2011 | TIG/ACT/01/2000&EXT Study Group; 13 centers in Belgium, the Netherlands, Germany, and Croatia | First enrolment 21 Feb 2002; 60-month completion by 1 Dec 2009 | Age 18–50 years; single symptomatic ICRS grade III/IV femoral-condyle lesion (1–5 cm²) | CCI or MFx (randomized) | 118 randomized; 112 treated/analyzed (51 CCI; 61 MFx) | No overlap identified with another included report. | Within-group estimates and factor-by-treatment analyses retained according to the reported analysis; pooled treatment contrasts were not reclassified as prognostic factors. |
| Asik et al., 2008 | Istanbul University and Gaziosmanpasa University, Turkey; two-center cohort | Oct 1998–Jan 2006 | Focal grade IV full-thickness medial-femoral-condyle lesion; stable knee without concomitant pathology | MFx | 90 patients | No overlap identified with another included report. | Counted as an independent cohort. |
| Kreuz et al., 2006a | Albert Ludwig University Freiburg, Germany | 1999–2002 | Age 18–55 years; single ICRS grade III full-thickness lesion (1–4 cm²); no relevant malalignment, instability, or meniscal pathology | MFx | 85 treated; 70 included | Confirmed same cohort as Kreuz 2006b: identical institution, period, eligibility, treatment, sample, follow-up schedule, and MRI–clinical correlation. | Treated as one independent cohort. This report was used for lesion-location and MRI results; duplicate estimates were counted once. |
| Kreuz et al., 2006b | Albert Ludwig University Freiburg, Germany | 1999–2002 | Age 18–55 years; single ICRS grade III full-thickness lesion (1–4 cm²); no relevant malalignment, instability, or meniscal pathology | MFx | 85 treated; 70 included | Confirmed same cohort as Kreuz 2006a. | Used for the age-focused analysis only where it provided the more detailed report; not counted independently from Kreuz 2006a. |
| Steadman et al., 2003 | Steadman-Hawkins Sports Medicine Foundation, Vail, Colorado, USA | 1981–1991 | Traumatic full-thickness chondral defect; age ≤45 years; no meniscal or ligament injury or chronic degenerative arthritis | MFx | 72 patients/75 knees eligible; 68 patients/71 knees analyzed | No overlap identified with another included report. | Counted as an independent cohort. |
| Koch et al., 2026 | Heidelberg University Hospital, Germany; single-center institutional database | 2011–2015 | Age >16 years; symptomatic unifocal ICRS grade III/IV knee defect; M-ACI; minimum 10-year follow-up | M-ACI | 112 patients | Overlap with Seiferth 2022 cannot be excluded because the German registry is multicenter and overlaps calendar time; participant-level linkage was unavailable. | Reported in the long-term PASS stratum and not interpreted as independent replication of the registry analysis for a shared factor–outcome–time stratum. |
| Philippe et al., 2026 | Lausanne University Hospital, Switzerland; prospective interventional cohort | 2017–2024 | Adults receiving second-generation ACI for symptomatic focal chondral or osteochondral knee defects; complete manufacturing and outcome data were required for each model. | Second-generation autologous chondrocyte implantation (ACI) | 67 patients | No material overlap identified with another included report. | Independent cohort; manufacturing-factor evidence retained at the reported mid-term horizon. |
| Tierney et al., 2025 | Robert Jones and Agnes Hunt Orthopaedic Hospital/OsCell database, UK | 1996–2020 | Knee ACI with at least one eligible postoperative Lysholm score before a second major procedure. | Autologous chondrocyte implantation (ACI) | 306 patients | No material overlap identified with another included report. | Independent longitudinal ACI cohort; short-term level and long-term slope estimates kept separate. |
| Cook et al., 2025 | Missouri Orthopaedic Institute, USA; prospective registry | 2016–2019 | Primary multisurface OCA performed ≥5 years before analysis; failures included regardless of final follow-up. | Osteochondral allograft transplantation (OCA/OCAT) | 184 patients/186 knees | No material overlap identified with another included report. | Independent report. Baseline patient/procedure factors retained; postoperative rehabilitation adherence excluded from the baseline prognostic synthesis. |
| Yazdi et al., 2025 | Midwest Orthopaedics at Rush, Chicago, USA | Jan 2003–Jan 2018 | Adults undergoing primary knee OCA with ≥5-year follow-up; concomitant procedures were allowed. | Primary osteochondral allograft transplantation (OCA) | 235 patients | Same institution and overlapping years as Rush MFx reports, but a different index procedure; no same-procedure overlap identified. | Kept separate from Rush MFx cohorts; multivariable threshold-outcome estimates retained. |
| Wang et al., 2025 | Scripps Clinic, La Jolla, USA; single-surgeon registry | 1997–2021 | Focal chondral/osteochondral lesions treated with OCA and ≥2-year follow-up. | Osteochondral allograft transplantation (OCA) | 527 patients/560 knees | No material overlap identified with another included report. | Independent OCA registry; multivariable failure estimates prioritized. |
| Ebert et al., 2025 | Perth, Western Australia; longitudinal MACI cohort | NR | MACI to medial or lateral femoral condyle with preoperative and 10-year clinical/MRI review. | Matrix-induced autologous chondrocyte implantation (MACI) | 96 patients | No material overlap identified with another included report. | Independent long-term structural-outcome cohort. |
| Weishorn et al., 2024 | Heidelberg University Hospital, Germany | Jan 2012–Dec 2018 | Symptomatic focal cartilage defects treated with MACI; 150 treated patients were contacted for long-term review. | Matrix-induced autologous chondrocyte implantation (MACI) | 103 patients | Highly likely partial overlap with Koch et al., 2026 (same institution, procedure and overlapping 2012–2015 recruitment); participant linkage unavailable. | Retained for long-term KOOS/MOCART estimates; not counted as independent replication of Koch for a shared factor–outcome–time unit. |
| Sato et al., 2024 | University of California, San Francisco, USA | 2014–2020 | Cartilage restoration with an available preoperative MRI and minimum 2-year PROM follow-up. | Mosaicplasty, OCA or MACI | 40 patients | No material overlap identified with another included report. | Independent mixed-procedure evidence; correlations retained descriptively and not assigned to an individual procedure. |
| Bumberger et al., 2023 | German Cartilage Registry (KnorpelRegister DGOU); multicenter | Registry period NR in report | Isolated cartilage restoration for focal knee lesions; bipolar and unipolar groups propensity matched. | ACI, BMS, osteochondral transfer and other restoration procedures | 238 matched patients | Probable participant overlap with Seiferth et al., 2022 (same German Cartilage Registry and overlapping procedure mix). | Retained only for the unique propensity-matched bipolar-versus-unipolar analysis; shared registry factors do not constitute independent replication. |
| Byrne et al., 2023 | PearlDiver M151Ortho claims database, USA | 2010–2020 | Primary OCA with continuous claims follow-up and no prior salvage procedure. | Osteochondral allograft transplantation (OCA) | 6,391 patients | Shares the PearlDiver vendor and calendar period with Gillinov et al., 2022, but uses a different dataset and index procedure. | Retained as a separate OCA claims cohort; cross-procedure concordance is not treated as replication. |
| Kim et al., 2020 | Yonsei Sarang Hospital, Seoul, South Korea | Nov 2011–Jan 2015 | Knee OA treated with arthroscopic MSC implantation and minimum 5-year follow-up. | Adipose-derived MSC implantation on fibrin glue scaffold | 467 patients/483 knees | Highly likely extended/overlapping Yonsei cohort relative to Koh 2014 and Kim 2015; exact participant linkage was not reported. | Retained for unique long-term Cox survival estimates; not treated as independent replication of earlier short-/mid-term Yonsei findings. |
| Merkely et al., 2021 | Brigham and Women's Hospital/Harvard cartilage practice, USA | NR | Patients undergoing ACI or OCA with available comorbidity and failure data. | Autologous chondrocyte implantation or osteochondral allograft transplantation | 368 patients (ACI 209; OCA 159) | No material overlap identified with another included report. | Independent report. ACI multivariable estimates retained; OCA findings remained unadjusted because no factor entered a multivariable model. |
| Wright-Chisem et al., 2021 | Hospital for Special Surgery, New York, USA | NR | Knee OCA with minimum 2-year PROM follow-up and self-reported allergy data. | Osteochondral allograft transplantation (OCA) | 245 patients | Probable partial overlap with other HSS OCA registry reports; participant-level linkage unavailable. | Retained for the unique drug-allergy analysis only. |
| Gillinov et al., 2022 | PearlDiver M91Ortho claims database, USA | 2010–2020 | Age 20–59 years; primary ACI without previous cartilage procedure or arthroplasty; 5-year claims follow-up. | Autologous chondrocyte implantation (ACI) | 533 patients | Shares the PearlDiver vendor and years with Byrne et al., 2023 but uses a different dataset and index procedure. | Retained as a separate ACI claims cohort. |
| Owusu-Akyaw et al., 2021 | Hospital for Special Surgery, New York, USA | Registry through 2017 | Femoral OCA with complete baseline SF-36/IKDC and 2-year IKDC questionnaires. | Femoral osteochondral allograft transplantation (OCA) | 91 patients | Likely subset of the HSS OCA registry used in records 333–334; exact participant linkage was not reported. | Preferred conventional adjusted OR/CI source for baseline PCS/MCS and defect size versus 2-year IKDC MCID. |
| Ramkumar et al., 2021a | Hospital for Special Surgery registry, New York, USA (analysis also involved Cleveland Clinic investigators) | Procedures before May 1, 2018 | Fresh knee OCA for up to two symptomatic Outerbridge grade 4 lesions with 1- or 2-year outcome follow-up. | Osteochondral allograft transplantation (OCA) | 185 patients | Confirmed same 185-patient cohort as Ramkumar et al., 2021b (record 334). | Retained for unique mental-health, pain-catastrophizing and activity-demand features; shared PROM/demographic associations counted once. |
| Ramkumar et al., 2021b | Hospital for Special Surgery registry, New York, USA (analysis also involved Cleveland Clinic investigators) | Procedures before May 1, 2018 | Same OCA eligibility cohort as Ramkumar et al., 2021a; 2-year outcomes and preoperative MRI required. | Osteochondral allograft transplantation (OCA) | 185 patients (153 at 2 years) | Confirmed same 185-patient cohort as Ramkumar et al., 2021a (record 333). | Retained for unique imaging/malalignment features; shared PROM/demographic associations counted once. |
| ACI, autologous chondrocyte implantation; CCI, characterized chondrocyte implantation; ICRS, International Cartilage Repair Society; KL, Kellgren–Lawrence; M-ACI, matrix-associated autologous chondrocyte implantation; MFx, microfracture; MSC, mesenchymal stem cell; NR, not reported; OA, osteoarthritis; PASS, patient acceptable symptom state; SVF, stromal vascular fraction. Confirmed overlap required direct evidence in the primary reports or agreement of institution, recruitment period, eligibility, treatment, sample, and follow-up. Possible overlap denotes shared data sources and overlapping periods without participant-level linkage. Reported sample sizes should not be interpreted as unique participant counts. For confirmed or possible overlap, only one report was allowed to contribute to a given prognostic factor–outcome–time-point synthesis. Reports from overlapping cohorts were retained when they contributed unique eligible prognostic-factor, outcome, or follow-up information; cohort overlap alone was not an automatic exclusion criterion. | | | | | | | |

**Supplementary Table 3.** Reports excluded after full-text eligibility assessment and primary reasons for exclusion.

| **Report** | **Primary reason for exclusion** | **Eligibility detail** |
| --- | --- | --- |
| Ota et al. (2024). Prognostic Factors in Intra-articular Platelet-Rich Plasma Treatment for Knee Osteoarthritis: A Comparative Analysis of Responders and Nonresponders. Cureus 16:e57645. doi:10.7759/cureus.57645. | Irrelevant procedure | Intra-articular platelet-rich plasma injection rather than a cartilage-repair procedure within the review scope. |
| Oo et al. (2023). Inflammatory ultrasound features as prognostic factors of pain and functional outcomes following intra-articular platelet-rich plasma in knee osteoarthritis. Int J Rheum Dis 26:1540–1546. doi:10.1111/1756-185x.14781. | Irrelevant procedure | Intra-articular platelet-rich plasma injection rather than marrow stimulation or operative biologic cartilage repair. |
| Smith et al. (2023). Derivation of a clinical decision rule for a bone marrow aspirate concentrate injection in knee osteoarthritis. Regen Med 18:461–470. doi:10.2217/rme-2023-0014. | Irrelevant procedure | Intra-articular bone marrow aspirate concentrate injection rather than surgical implantation or cartilage repair. |
| Guarino et al. (2023). Long-Term Survival and Predictors of Failure of Opening Wedge High Tibial Osteotomy. Orthop Surg 15:1002–1007. doi:10.1111/os.13674. | Irrelevant procedure | High tibial osteotomy was the index procedure; the outcome was survival to arthroplasty rather than outcome after an eligible cartilage-repair procedure. |
| Sofu et al. (2022). Lesion size and varus malalignment are the major determinants leading to poorer clinical outcomes after combined microfracture treatment for focal cartilage lesions during anterior cruciate ligament reconstruction. Arch Orthop Trauma Surg 142:1941–1949. doi:10.1007/s00402-021-04138-x. | Irrelevant procedure | Microfracture was performed only with anterior cruciate ligament reconstruction, so the reported outcome could not be attributed to the cartilage-repair procedure in the prespecified context. |
| Miller et al. (2007). Patient satisfaction after medial opening high tibial osteotomy and microfracture. J Knee Surg 20:129–133. doi:10.1055/s-0030-1248031. | Irrelevant procedure | High tibial osteotomy was the principal intervention and microfracture was concomitant; the reported predictors addressed the combined osteotomy procedure. |
| Lee et al. (2026). Clinical outcomes after medial open-wedge high tibial osteotomy are not affected by joint space in extension and flexion on standing radiographs. Arch Orthop Trauma Surg 146(1). doi:10.1007/s00402-026-06197-4. | Irrelevant procedure | High tibial osteotomy was the index procedure, without an eligible cartilage-repair procedure within the review scope. |
| Dias Abeyagunawardene et al. (2025). Knee-to-Talus osteochondral grafting for talar lesions: Mid-term outcomes of the OATS procedure. Foot Ankle Surg Tech Rep Cases 5(3):100544. doi:10.1016/j.fastrc.2025.100544. | Irrelevant procedure | The treated cartilage lesion and postoperative outcome involved the talus; the knee served only as the osteochondral graft donor site. |
| Çakar et al. (2025). Predictors of Donor Site Morbidity Following Osteochondral Graft Harvesting from the Healthy Knee using Lysholm Knee Score. Istanbul Med J 26:73–80. doi:10.4274/imj.galenos.2025.70120. | Irrelevant procedure | The index cartilage-repair procedure treated talar lesions, and the knee analysis addressed donor-site morbidity rather than outcome after knee cartilage repair. |
| Gille et al. (2024). Patient-Reported Outcome Measures in the Repair of Chondral Defects: How Well Do the Scores Relate to Each Other? Surg Technol Int 45. PMID:39374580. | No eligible prognostic-factor analysis or extractable postoperative association | The analysis assessed correlations among postoperative patient-reported outcome measures rather than a baseline prognostic factor associated with a postoperative outcome. |
| Faber et al. (2022). Factors correlating with patients' satisfaction after undergoing cartilage repair surgery—data from the German Cartilage Registry (KnorpelRegister DGOU). Int Orthop 46:457–464. doi:10.1007/s00264-021-05274-0. | No eligible prognostic-factor analysis or extractable postoperative association | The principal analyses correlated postoperative satisfaction with postoperative scores; baseline factors were reported collectively without extractable procedure-specific factor–outcome estimates. |
| DiBartola et al. (2021). Predictors of poor pre-operative psychological status among patients with cartilage defects. Knee 33:11–16. doi:10.1016/j.knee.2021.08.026. | No eligible prognostic-factor analysis or extractable postoperative association | The outcomes were preoperative psychological measures; no postoperative cartilage-repair outcome was evaluated. |
| Gobbi et al. (2015). Matrix-Induced Autologous Chondrocyte Implantation versus Multipotent Stem Cells for the Treatment of Large Patellofemoral Chondral Lesions: A Nonrandomized Prospective Trial. Cartilage 6:82–97. doi:10.1177/1947603514563597. | No eligible prognostic-factor analysis or extractable postoperative association | The study primarily compared two treatments and did not provide an extractable baseline prognostic-factor effect estimate for the eligible synthesis. |
| Gille et al. (2013). Outcome of Autologous Matrix Induced Chondrogenesis (AMIC) in cartilage knee surgery: data of the AMIC Registry. Arch Orthop Trauma Surg 133:87–93. doi:10.1007/s00402-012-1621-5. | No eligible prognostic-factor analysis or extractable postoperative association | The report described postoperative outcome after AMIC without an eligible baseline factor–outcome analysis. |
| Zaslav et al. (2009). A prospective study of autologous chondrocyte implantation in patients with failed prior treatment for articular cartilage defect of the knee: results of the Study of the Treatment of Articular Repair (STAR) clinical trial. Am J Sports Med 37:42–55. doi:10.1177/0363546508322897. | No eligible prognostic-factor analysis or extractable postoperative association | The report focused on treatment effectiveness and prior-treatment subgroups and did not provide an extractable baseline prognostic-factor effect estimate for the eligible synthesis. |
| Ramappa et al. (2007). Magnetic resonance imaging to assess knee cartilage repair tissue after microfracture of chondral defects. J Knee Surg 20:228–234. doi:10.1055/s-0030-1248048. | No eligible prognostic-factor analysis or extractable postoperative association | The analysis evaluated postoperative MRI diagnostic performance against second-look arthroscopy, not a baseline prognostic factor associated with postoperative outcome. |
| Moon et al. (2026). MOCART 2.0 score of 60 or greater measured at 1 year post-operatively predicts favourable clinical outcomes after surgical repair of tibiofemoral cartilage lesions. Knee Surg Sports Traumatol Arthrosc 34:3247–3256. doi:10.1002/ksa.70086. | No eligible prognostic-factor analysis or extractable postoperative association | The principal predictor was the MOCART 2.0 score measured 1 year after surgery, rather than a factor defined at or before treatment. |
| Knapik et al. (2026). Patient and Chondral Defect–Specific Characteristics and Associated Matrix-Induced Autologous Chondrocyte Implantation Conversion Rates Following Biopsy. Orthop J Sports Med 14:23259671251415555. doi:10.1177/23259671251415555. | No eligible prognostic-factor analysis or extractable postoperative association | The outcome was conversion from cartilage biopsy to subsequent MACI, not a clinical or structural postoperative outcome after an eligible cartilage-repair procedure. |
| Gersing et al. (2020). Cartilage T2 Relaxation Times and Subchondral Trabecular Bone Parameters Predict Morphological Outcome After Matrix-Associated Autologous Chondrocyte Implantation With Autologous Bone Grafting. Am J Sports Med 48:3573–3585. doi:10.1177/0363546520965987. | No eligible prognostic-factor analysis or extractable postoperative association | The principal predictors were MRI cartilage and subchondral-bone measurements obtained 3 months after surgery, rather than baseline prognostic factors. |
| Brown et al. (2020). Osteochondritis Dissecans Lesion Location Is Highly Concordant With Mechanical Axis Deviation. Am J Sports Med 48:871–875. doi:10.1177/0363546520905567. | No eligible prognostic-factor analysis or extractable postoperative association | The cross-sectional analysis related preoperative alignment to lesion location and size and did not evaluate a postoperative cartilage-repair outcome. |
| van Tuijn et al. (2023). Prognostic Factors for the Clinical Outcome after Microfracture Treatment of Chondral and Osteochondral Defects in the Knee Joint: A Systematic Review. Cartilage 14:5–16. doi:10.1177/19476035221147680. | Ineligible study design | Systematic review rather than an original prognostic-factor study. |
| Migliorini et al. (2023). Prognostic factors for the management of chondral defects of the knee and ankle joint: a systematic review. Eur J Trauma Emerg Surg 49:723–745. doi:10.1007/s00068-022-02155-y. | Ineligible study design | Systematic review rather than an original prognostic-factor study. |
| Toyooka et al. (2023). Return to Pivoting Sports after Cartilage Repair Surgery of the Knee: A Scoping Review. Cartilage 14:17–25. doi:10.1177/19476035221141416. | Ineligible study design | Scoping review rather than an original prognostic-factor study. |
| Jeuken et al. (2021). A Systematic Review of Focal Cartilage Defect Treatments in Middle-Aged Versus Younger Patients. Orthop J Sports Med 9:23259671211031244. doi:10.1177/23259671211031244. | Ineligible study design | Systematic review rather than an original prognostic-factor study. |
| Smoak et al. (2019). Systematic Review of Patient Outcomes and Associated Predictors After Microfracture in the Patellofemoral Joint. J Am Acad Orthop Surg Glob Res Rev 3:e10.5435/JAAOSGlobal-D-19-00151. doi:10.5435/JAAOSGlobal-D-19-00151. | Ineligible study design | Systematic review rather than an original prognostic-factor study. |
| Bartkoski et al. (2026). Impact of Donor-Recipient Sex-Mismatch on Graft Failure in Osteochondral Allograft Procedures: A Systematic Review. Orthop J Sports Med 14:23259671251407241. doi:10.1177/23259671251407241. | Ineligible study design | Systematic review rather than an original prognostic-factor study. |
| Liu et al. (2025). Tissue Engineered Cartilage Repair Using Small-Incision Implantation of Decalcified Corticocancellous Bone Scaffold. Arthrosc Tech 14(4):103346. doi:10.1016/j.eats.2024.103346. | Ineligible study design | Technical note describing an implantation technique rather than an original prognostic-factor study. |
| Nimkingratana and Brittberg (2020). Returning to Work After Articular Cartilage Repair Intervention: A Systematic Review. Orthop J Sports Med 8:2325967120905526. doi:10.1177/2325967120905526. | Ineligible study design | Systematic review rather than an original prognostic-factor study. |
| Primary exclusion reasons: irrelevant procedure (n=9); no eligible prognostic-factor analysis or extractable postoperative association (n=11); and ineligible study design (n=8). Each report was assigned one primary reason, although some reports met more than one exclusion criterion. | | |

**Supplementary Table 4.** Summary of the surgical techniques reported in the included studies.

| Study | Reported surgical technique |
| --- | --- |
| Saad Berreta et al., 2026 | Primary arthroscopic microfracture was performed after debridement to stable cartilage margins, using a PowerPick or Steadman awl for subchondral perforation. A subset received BMAC augmentation. Rehabilitation was adapted to lesion location. |
| Koch et al., 2026 | Matrix-associated autologous chondrocyte implantation was performed for ICRS grade III/IV knee defects. Chondrocytes cultured on a biphasic collagen matrix were implanted through mini-open arthrotomy approximately 4 weeks after arthroscopic harvest and fixed with resorbable sutures and fibrin glue. |
| Kim et al., 2023 | Gluteal adipose tissue was processed to obtain SVF, with sterility, cell viability, and cell characteristics assessed before implantation. After arthroscopic debridement and subchondral drilling, the SVF was combined with fibrin glue and applied arthroscopically to the cartilage lesion. |
| Seiferth et al., 2022 | The registry analysis included autologous chondrocyte implantation procedures performed across participating centers; a single standardized operative technique was not specified in the report. |
| Wang et al., 2021 | Patients underwent either biphasic synthetic scaffold implantation or arthroscopic microfracture. Scaffold implantation used a mini-arthrotomy, preparation of host sites with trephine and coring instruments, and impaction of plugs flush with the surrounding cartilage. Microfracture involved debridement to stable margins, removal of calcified cartilage, and subchondral perforation. |
| Chahla et al., 2020 | Primary arthroscopic microfracture was performed by a single fellowship-trained surgeon. Additional standardized operative details were not reported. |
| Solheim et al., 2018 | Patients underwent either arthroscopic microfracture or mosaicplasty, selected according to surgeon preference and patient or lesion characteristics. Microfracture used debridement to stable margins and angled-awl subchondral perforation; mosaicplasty used osteochondral grafts harvested from the femoral-condyle periphery and transplanted to prepared recipient sites. |
| Kim et al., 2015a | After arthroscopic debridement, MSCs were implanted without marrow stimulation. In the local-adherent group, the cell suspension was applied directly to the defect; in the scaffold group, MSCs were combined with fibrin glue and applied arthroscopically after fluid evacuation. |
| Koh et al., 2014 | After arthroscopic debridement, adipose-derived MSCs were applied directly to the defect using a local-adherent technique after fluid evacuation and positioning the lesion upward. No microfracture, subchondral drilling, or abrasion arthroplasty was performed before implantation. |
| Vanlauwe et al., 2011 | Participants were randomized to CCI or microfracture. CCI used an open autologous chondrocyte implantation technique with periosteal coverage; microfracture used arthroscopic marrow stimulation. Both groups followed the same standardized rehabilitation program. |
| Asik et al., 2008 | After arthroscopic debridement to stable margins and removal of calcified cartilage, microfracture perforations were created with an awl from the defect periphery toward the center while preserving intervening subchondral bone. |
| Kreuz et al., 2006a | Microfracture was performed by two surgeons using debridement to stable margins and evenly distributed angled-awl perforations. Irrigation pressure was reduced to confirm bleeding, and a nonsuction intra-articular drain was placed. |
| Kreuz et al., 2006b | This overlapping report described the same two-surgeon microfracture protocol: arthroscopic debridement, angled-awl subchondral perforation, confirmation of bleeding after reducing irrigation pressure, and placement of a nonsuction intra-articular drain. |
| Philippe et al., 2026 | Cartilage biopsy, GMP expansion and second-generation ACI; initial chondrocyte yield, gene expression, culture confluence and population-doubling time were recorded before implantation. |
| Tierney et al., 2025 | First- and later-generation ACI using periosteal or collagen patches; implanted-cell number and passage were recorded. |
| Cook et al., 2025 | Single- or multisurface OCA, including bipolar constructs; meniscal allograft and alignment/ligament procedures as indicated. |
| Yazdi et al., 2025 | Fresh primary OCA with lesion-specific grafting; concomitant meniscal, alignment and ligament procedures as indicated. |
| Wang et al., 2025 | Fresh OCA using plug or shell techniques according to lesion size and location. |
| Ebert et al., 2025 | Third-generation MACI after biopsy and cell expansion; membrane secured with fibrin glue. |
| Weishorn et al., 2024 | Third-generation MACI with a cell-seeded collagen membrane fixed using fibrin glue. |
| Sato et al., 2024 | Mosaicplasty, OCA or MACI according to clinical indication; preoperative MRI graded with WORMS. |
| Bumberger et al., 2023 | Registry-based cartilage restoration; ACI predominated, with BMS, osteochondral transfer and combined approaches also represented. |
| Byrne et al., 2023 | Claims-coded OCA; operative graft details were unavailable. |
| Kim et al., 2020 | Autologous adipose-derived MSCs implanted in fibrin glue after arthroscopic defect preparation. |
| Merkely et al., 2021 | ACI or OCA using standard institutional techniques; estimates were reported separately by procedure. |
| Wright-Chisem et al., 2021 | Fresh OCA; number of self-reported drug allergies recorded preoperatively. |
| Gillinov et al., 2022 | Claims-coded primary ACI; graft/manufacturing details unavailable. |
| Owusu-Akyaw et al., 2021 | Fresh femoral OCA; baseline SF-36 PCS/MCS evaluated as predictors. |
| Ramkumar et al., 2021a | Fresh OCA; seven machine-learning architectures with SHAP feature interpretation. |
| Ramkumar et al., 2021b | Fresh OCA; preoperative MRI and patient variables analyzed with seven machine-learning architectures and SHAP. |
| Steadman et al., 2003 | Microfracture used three arthroscopic portals, debridement to stable perpendicular margins, removal of the calcified cartilage layer, and angled-awl perforations from the periphery toward the center while preserving subchondral bone bridges. Irrigation pressure was reduced to confirm bleeding; no drain was placed. |

**Supplementary Table 5.** Study-level eligibility, recruitment, lesion, concomitant-procedure, and follow-up details.

| **Study** | **Key inclusion/exclusion criteria** | **Recruitment period** | **Lesion pathology** | **Concomitant procedures** | **Follow-up completeness** |
| --- | --- | --- | --- | --- | --- |
| Saad Berreta et al., 2026 | Symptomatic tibiofemoral cartilage defect after failed nonoperative care; primary arthroscopic MFx; minimum 5-year follow-up. Revision MFx, previous cartilage-preserving surgery, concomitant ligament injury, osteotomy-requiring malalignment, repair-requiring meniscal tear, subsequent ipsilateral surgery, and incomplete final PROMs were excluded. | Jan 2013–Sep 2018 | Symptomatic tibiofemoral cartilage defect treated with primary MFx. | Major ligament, osteotomy, and meniscal-repair procedures were excluded. BMAC was used as an adjunct in a subset. | 103 patients were eligible for 5-year assessment; 70 had complete final PROMs (68% compliance). |
| Koch et al., 2026 | Age >16 years; symptomatic unifocal ICRS grade III/IV knee defect; failed nonoperative care; M-ACI; minimum 10-year follow-up. KL grade >2, >1/3 meniscal deficit, instability, untreated malalignment >3°, bipolar lesions, specified previous hip/ankle surgery, consent withdrawal, and conversion to arthroplasty were excluded. | 2011–2015 | Unifocal symptomatic ICRS grade III/IV articular cartilage defect of the knee. | Corrective osteotomy was permitted/performed where required; no concomitant ACL or collateral-ligament reconstruction was reported. | 112 patients were evaluated at ≥10 years; 14 were excluded at long-term assessment and 3 were lost to follow-up. |
| Kim et al., 2023 | Symptomatic knee OA unresponsive to nonoperative care. Previous surgery, knee instability, varus/valgus malalignment, rheumatoid arthritis, haemophilia, active infection, and other specified disease were excluded. | Sep 2019–Apr 2021 | OA-associated cartilage lesions assessed clinically and by 12-month MRI; femoral-condyle and trochlear lesions were represented. | Subchondral drilling was an integral component of SVF implantation; no additional concomitant surgical procedure was reported. | Of 62 qualified patients, 14 dropped out and 5 were lost; 43 completed the analysis (69.4%). |
| Seiferth et al., 2022 | Registry eligibility required adulthood, operative cartilage treatment, consent, and an email address. The present analysis selected knee ACI recipients with intact menisci and compared previous-surgery exposure using propensity-score matching. | Registry initiated 2013; data through Dec 2020 | Knee chondral defects treated with ACI; defect grade, location, cause, and corresponding-surface status varied. | Intact menisci were required for the analyzed cohort. Other concomitant index procedures were not reported at study level. | Available-case data were reported at 6, 12, 24, and 36 months; denominators varied by time point. No single overall completion rate was reported, and substantial registry loss to follow-up was acknowledged. |
| Wang et al., 2021 | Skeletally mature; one symptomatic Outerbridge grade III/IV femoral-condyle or trochlear lesion, 1–6 cm²; no major osseous deficiency; minimum 2-year follow-up. Generalized OA, inflammatory arthritis, osteonecrosis, multiple lesions, instability, major meniscal deficiency, patellar lesions, maltracking, and osteotomy-requiring malalignment were excluded. | Registry screening 1999–2014 | Single symptomatic grade III/IV femoral-condyle or trochlear chondral/osteochondral lesion. | Concomitant osteotomy, ligament reconstruction, and meniscal transplantation were excluded; concurrent meniscal tear was recorded as a model candidate. | Among 490 identified patients, 132 were included (66/group) after exclusions. At 2 years, PROM responses were available for 54 scaffold (82%) and 42 MFx (64%) patients; at 5 years, 13 (20%) and 14 (21%), respectively. |
| Chahla et al., 2020 | Symptomatic knee pain after failed nonoperative management; primary MFx; minimum 2-year follow-up. Revision MFx, previous cartilage-preserving treatment, traumatic ipsilateral knee injury, femoral-notch MFx, absent baseline PROMs, and inadequate follow-up were excluded. | Jan 2004–Jan 2017 | Symptomatic focal knee cartilage defects treated with primary MFx; lesion size, number, and location varied. | Concomitant procedures were recorded but were missing for >30% of the cohort and were not analyzed. | Of 521 queried patients, 27 lacked minimum 2-year follow-up and 288 lacked baseline PROMs; 206 were analyzed. |
| Solheim et al., 2018 | Age ≤60 years; symptomatic focal full-thickness chondral lesion treated with MFx or mosaicplasty. Joint-space width <4 mm, malalignment >5°, previous/concurrent realignment surgery, instability, and inability to follow rehabilitation were excluded. | 1998–2003 | Focal full-thickness lesions; single or multiple lesions and normal or mildly degenerative surrounding cartilage were represented. | Previous or concomitant partial medial meniscectomy was present in 46/205 cases (22%). | All 205 patients had at least one follow-up of ≥1 year (100%); the most recent follow-up was used (median 14 years, range 1–18). |
| Kim et al., 2015 | Isolated full-thickness cartilage lesion in KL grade 1–2 OA after ≥3 months of nonsurgical treatment. Previous surgery, multiple lesions, instability, malalignment ≥5°, metabolic arthritis, infection, and large meniscal tear were excluded. | Oct 2010–Apr 2012 | Isolated full-thickness OA-associated cartilage lesion treated with MSC implantation with or without fibrin-glue scaffold. | Large meniscal tears and previous surgery were excluded; no marrow-stimulation procedure was performed. Other concomitant index procedures were not reported. | Of 88 eligible patients/93 knees, 54 patients/56 knees underwent second-look arthroscopy and formed the analyzed cohort. |
| Koh et al., 2014 | Isolated full-thickness cartilage lesion in KL grade 1–2 OA after ≥3 months of nonoperative care; primary cases only. Multiple lesions, previous surgery, instability, malalignment ≥5°, metabolic arthritis, infection, and large meniscal tear were excluded. | Oct 2010–Sep 2011 | Isolated full-thickness OA-associated cartilage lesion treated with local-adherent adipose-derived MSC implantation. | Large meniscal tears and previous surgery were excluded; no marrow-stimulation procedure was performed. Other concomitant index procedures were not reported. | Of 56 patients/60 knees treated, 35 patients/37 knees underwent second-look arthroscopy and were included in the prognostic analysis. |
| Vanlauwe et al., 2011 | Age 18–50 years; single symptomatic ICRS grade III/IV femoral-condyle lesion, 1–5 cm²; participation in the rehabilitation and follow-up program. Detailed criteria were reported in the preceding trial publication; radiographic OA was excluded. | First enrolment Feb 21, 2002; 60-month completion by Dec 1, 2009 | Single symptomatic ICRS grade III/IV femoral-condyle lesion randomized to CCI or MFx. | Associated ACL, meniscal, or other lesions were reported at baseline; corresponding concomitant procedures were not separately quantified. | 118 were randomized and 112 treated. Sixty-month data were provided by 43/51 CCI patients (84%) and 45/61 MFx patients (74%); last observation carried forward was used for documented withdrawals. |
| Asik et al., 2008 | Focal full-thickness medial-femoral-condyle lesion in a stable knee. Patients with ligament/meniscal or other concomitant pathology, patellofemoral problems, multiple locations, or subsequent secondary surgery were excluded. | Oct 1998–Jan 2006 | Focal full-thickness medial-femoral-condyle lesion; weight-bearing and non-weight-bearing locations were represented. | No concomitant pathology was permitted in the analyzed cohort. | Of 246 treated patients, 28 were lost to regular follow-up; after other eligibility exclusions, 90 were analyzed (mean follow-up 68 months). |
| Kreuz et al., 2006a | Age 18–55 years; one-compartment ICRS grade III full-thickness lesion, 1–4 cm². Recent trauma, malalignment, motion restriction, instability, meniscal pathology, recent injections/arthroscopy, and other specified conditions were excluded. | 1999–2002 | Single ICRS grade III lesion in a femoral-condyle, tibial, trochlear, or patellar location. | Relevant meniscal pathology, ligament instability, and malalignment were excluded; no concomitant procedure was reported. | Of 85 treated patients, 70 met the criteria and were evaluated through 36 months; loss after inclusion was not separately reported. |
| Kreuz et al., 2006b | Age 18–55 years; one-compartment ICRS grade III full-thickness lesion, 1–4 cm². Acute trauma, malalignment, motion restriction, instability, meniscal pathology, osteochondral defect, and recent OA treatment/arthroscopy were excluded. | 1999–2002 | Same MFx cohort as Kreuz et al. 2006a; single ICRS grade III lesion across knee compartments. | Relevant meniscal pathology, ligament instability, and malalignment were excluded; no concomitant procedure was reported. | Same 70/85 cohort as Kreuz et al. 2006a, evaluated through 36 months; not an independent cohort. |
| Steadman et al., 2003 | Age ≤45 years; traumatic full-thickness chondral defect; no meniscal or ligament injury. Degenerative joint-space narrowing and knees requiring treatment for ACL or meniscal tears were excluded. | 1981–1991 | Traumatic full-thickness chondral defect; acute and chronic lesions were represented. | No associated meniscal or ligament procedure was permitted. | 72 patients/75 knees met eligibility; 71 knees were available at final follow-up (95%; mean 11.3 years). |
| Philippe et al., 2026 | Adults receiving second-generation ACI for symptomatic focal chondral or osteochondral knee defects; complete manufacturing and outcome data were required for each model. | 2017–2024 | Focal chondral/osteochondral defects; mean defect area 5.4 cm². | Concomitant procedures were reported in the cohort; factor models focused on cell-manufacturing variables. | 67 enrolled; complete-case regression generally n=53 (IKDC n=54; MOCART n=45). |
| Tierney et al., 2025 | Knee ACI with at least one eligible postoperative Lysholm score before a second major procedure. | 1996–2020 | Predominantly ICRS grade 3–4 focal defects; single or multiple sites. | Concurrent procedures were retained and modeled; prior microfracture, scaffold type and cell variables were evaluated. | 306/427 procedures analyzed; response declined with time and only 23 patients supplied ≥10 annual scores; missing baseline values were imputed. |
| Cook et al., 2025 | Primary multisurface OCA performed ≥5 years before analysis; failures included regardless of final follow-up. | 2016–2019 | Unipolar, multisurface, patellofemoral bipolar and tibiofemoral bipolar osteochondral disease. | Ligament reconstruction, osteotomy and meniscal allograft transplantation were allowed. | 186 cases/184 patients analyzed; the eligible denominator and loss-to-follow-up proportion were incompletely reported. |
| Yazdi et al., 2025 | Adults undergoing primary knee OCA with ≥5-year follow-up; concomitant procedures were allowed. | Jan 2003–Jan 2018 | Focal chondral/osteochondral defects at femoral condyle, trochlea or patella. | Meniscal allograft, osteotomy and other major concomitant procedures were modeled. | 235 patients with minimum 5-year PROM data; source-database denominator was not fully reported. |
| Wang et al., 2025 | Focal chondral/osteochondral lesions treated with OCA and ≥2-year follow-up. | 1997–2021 | OCD, traumatic or degenerative chondral injury, fracture, avascular necrosis or osteoarthritis. | Prior surgery and multiple grafts were permitted; total graft area and location were evaluated. | 560 knees with minimum 2-year follow-up; loss before eligibility was not fully quantified. |
| Ebert et al., 2025 | MACI to medial or lateral femoral condyle with preoperative and 10-year clinical/MRI review. | NR | ICRS grade III–IV tibiofemoral lesions. | Prior knee procedures were eligible and explicitly modeled. | 96 patients with 10-year MRI review; derivation from the earlier eligible cohort is shown in the source flowchart. |
| Weishorn et al., 2024 | Symptomatic focal cartilage defects treated with MACI; 150 treated patients were contacted for long-term review. | Jan 2012–Dec 2018 | Predominantly ICRS grade III–IV focal defects; femorotibial and patellofemoral sites. | Previous knee surgery was modeled; relevant concomitant correction was performed as indicated. | 103/150 had complete clinical follow-up; 75 had current MRI and 59 entered the multivariable KOOS model. |
| Sato et al., 2024 | Cartilage restoration with an available preoperative MRI and minimum 2-year PROM follow-up. | 2014–2020 | Focal cartilage lesions across knee compartments with whole-joint MRI abnormalities. | Mixed procedures and compartments; potential confounders could not be modeled because of sample size. | 40 patients analyzed; source-cohort denominator and loss to follow-up were incompletely reported. |
| Bumberger et al., 2023 | Isolated cartilage restoration for focal knee lesions; bipolar and unipolar groups propensity matched. | Registry period NR in report | Full-thickness unipolar versus bipolar opposing-surface lesions. | Procedure type varied; treatment of the opposing lesion was common in the bipolar group. | 119 matched patients per group; follow-up availability declined substantially by 24–36 months. |
| Byrne et al., 2023 | Primary OCA with continuous claims follow-up and no prior salvage procedure. | 2010–2020 | Claims-defined OCA population; lesion morphology unavailable. | Prior or concomitant bony realignment was evaluated; comorbidities were claims-defined. | 6,391 claims-defined patients; continuous enrollment criteria reduce observed attrition but clinical detail is unavailable. |
| Kim et al., 2020 | Knee OA treated with arthroscopic MSC implantation and minimum 5-year follow-up. | Nov 2011–Jan 2015 | OA-associated femoral-condyle lesions, including bipolar kissing lesions. | Alignment correction and lesion treatment were performed according to the institutional protocol. | 467 patients/483 knees with minimum 5-year follow-up; survival analysis extended to 9 years. |
| Merkely et al., 2021 | Patients undergoing ACI or OCA with available comorbidity and failure data. | NR | Focal cartilage defects; procedure-specific lesion size differed. | Osteotomy, meniscal allograft and ligament reconstruction were permitted and reported. | 209 ACI and 159 OCA patients analyzed; source denominator and attrition were incompletely reported. |
| Wright-Chisem et al., 2021 | Knee OCA with minimum 2-year PROM follow-up and self-reported allergy data. | NR | Focal chondral/osteochondral defects. | Concomitant procedures were allowed and described. | 245 patients analyzed at minimum 2 years; source denominator and exact overlap with other HSS reports were not reported. |
| Gillinov et al., 2022 | Age 20–59 years; primary ACI without previous cartilage procedure or arthroplasty; 5-year claims follow-up. | 2010–2020 | Claims-defined focal full-thickness chondral defects; morphology unavailable. | Realignment within one year before or at ACI was modeled. | 533 continuously observed patients; claims-based clinical detail was limited. |
| Owusu-Akyaw et al., 2021 | Femoral OCA with complete baseline SF-36/IKDC and 2-year IKDC questionnaires. | Registry through 2017 | Focal femoral chondral/osteochondral defects. | Prior surgery and concomitant ACL reconstruction were modeled. | 91/141 patients had complete baseline and 2-year data. |
| Ramkumar et al., 2021a | Fresh knee OCA for up to two symptomatic Outerbridge grade 4 lesions with 1- or 2-year outcome follow-up. | Procedures before May 1, 2018 | Symptomatic focal cartilage defects without substantial bone loss requiring shell graft/bone grafting. | Previous and concomitant procedures were included as candidate predictors. | 185 eligible; 135 (73%) and 153 (83%) had 1- and 2-year follow-up, respectively. |
| Ramkumar et al., 2021b | Same OCA eligibility cohort as Ramkumar et al., 2021a; 2-year outcomes and preoperative MRI required. | Procedures before May 1, 2018 | Outerbridge grade 4 focal defects; imaging variables included edema, lesion size, alignment and associated injury. | ACL/meniscal injury, prior surgery and number of OCA plugs were candidate predictors. | 185 eligible; 153 (83%) had 2-year follow-up. |
| ACI, autologous chondrocyte implantation; ACL, anterior cruciate ligament; BMAC, bone marrow aspirate concentrate; CCI, characterized chondrocyte implantation; ICRS, International Cartilage Repair Society; KL, Kellgren–Lawrence; M-ACI, matrix-associated autologous chondrocyte implantation; MFx, microfracture; MRI, magnetic resonance imaging; MSC, mesenchymal stem cell; NR, not reported; OA, osteoarthritis; PROM, patient-reported outcome measure; SVF, stromal vascular fraction. Follow-up completeness is reproduced from the reporting available in each primary report; no unreported denominator or completion rate was inferred. The two Kreuz reports describe the same cohort. | | | | | |

**Supplementary Table 6.** Methodological quality of the included studies assessed using the MINORS (Methodological Index for Non-Randomized Studies) criteria.

| Study | LOE | 1. A stated aim of the study | 2. Inclusion of consecutive patients | 3. Prospective collection of data | 4. Endpoint appropriate to the study aim | 5. Unbiased evaluation of endpoints | 6. Follow-up period appropriate to the major endpoint | 7. Loss to follow up not exceeding 5% | 8. Prospective calculation of the sample size | 9. A control group having the gold standard intervention | 10. Contemporary groups | 11. Baseline equivalence of groups | 12. Statistical analyses adapted to the study design | Total scores |
| --- | --- | --- | --- | --- | --- | --- | --- | --- | --- | --- | --- | --- | --- | --- |
| Saad Berreta et al., 2026 | III | 2 | 2 | 1 | 2 | 2 | 2 | 1 | 0 | - | - | - | - | 12 |
| Koch et al., 2026 | IV | 2 | 2 | 2 | 2 | 1 | 2 | 1 | 1 | - | - | - | - | 13 |
| Kim et al., 2023 | IV | 2 | 2 | 1 | 2 | 2 | 2 | 1 | 0 | - | - | - | - | 12 |
| Seiferth et al., 2022 | III | 2 | 2 | 2 | 2 | 1 | 2 | 1 | 0 | 2 | 2 | 2 | 2 | 20 |
| Wang et al., 2021 | III | 2 | 2 | 2 | 2 | 1 | 2 | 1 | 1 | 2 | 2 | 1 | 2 | 20 |
| Chahla et al., 2020 | IV | 2 | 2 | 2 | 2 | 2 | 1 | 2 | 0 | - | - | - | - | 13 |
| Solheim et al., 2018 | IV | 2 | 2 | 2 | 2 | 1 | 2 | 2 | 0 |  | - | - | - | 13 |
| Kim et al., 2015a | III | 2 | 2 | 1 | 2 | 1 | 2 | 1 | 1 | 2 | 2 | 2 | 2 | 20 |
| Koh et al., 2014 | IV | 2 | 2 | 1 | 2 | 1 | 2 | 1 | 1 | - | - | - | - | 12 |
| Asik et al., 2008 | IV | 2 | 2 | 1 | 2 | 2 | 2 | 1 | 0 | - | - | - | - | 12 |
| Kreuz et al., 2006a | IV | 2 | 2 | 2 | 2 | 1 | 2 | 2 | 0 | - | - | - | - | 13 |
| Kreuz et al., 2006b | IV | 2 | 2 | 2 | 2 | 1 | 2 | 2 | 0 | - | - | - | - | 13 |
| Steadman et al., 2003 | IV | 2 | 2 | 2 | 2 | 1 | 2 | 2 | 0 | - | - | - | - | 13 |
| Philippe et al., 2026 | IV | 2 | 1 | 2 | 2 | 1 | 2 | 0 | 0 | - | - | - | - | 10 |
| Tierney et al., 2025 | IV | 2 | 1 | 2 | 2 | 2 | 2 | 0 | 0 | - | - | - | - | 11 |
| Cook et al., 2025 | III | 2 | 1 | 2 | 2 | 1 | 2 | 0 | 0 | - | - | - | - | 10 |
| Yazdi et al., 2025 | III | 2 | 1 | 2 | 2 | 2 | 2 | 1 | 0 | - | - | - | - | 12 |
| Wang et al., 2025 | IV | 2 | 1 | 1 | 2 | 2 | 2 | 1 | 0 | - | - | - | - | 11 |
| Ebert et al., 2025 | III | 2 | 1 | 2 | 2 | 2 | 2 | 1 | 0 | - | - | - | - | 12 |
| Weishorn et al., 2024 | IV | 2 | 1 | 2 | 2 | 2 | 2 | 0 | 0 | - | - | - | - | 11 |
| Sato et al., 2024 | IV | 2 | 1 | 0 | 2 | 2 | 2 | 0 | 0 | - | - | - | - | 9 |
| Bumberger et al., 2023 | III | 2 | 1 | 2 | 2 | 2 | 2 | 0 | 0 | 2 | 2 | 2 | 2 | 19 |
| Byrne et al., 2023 | IV | 2 | 2 | 0 | 2 | 1 | 2 | 2 | 0 | - | - | - | - | 11 |
| Kim et al., 2020 | IV | 2 | 1 | 1 | 2 | 2 | 2 | 1 | 0 | - | - | - | - | 11 |
| Merkely et al., 2021 | III | 2 | 1 | 0 | 2 | 2 | 2 | 1 | 0 | - | - | - | - | 10 |
| Wright-Chisem et al., 2021 | III | 2 | 1 | 1 | 2 | 2 | 2 | 1 | 0 | - | - | - | - | 11 |
| Gillinov et al., 2022 | IV | 2 | 2 | 0 | 2 | 1 | 2 | 2 | 0 | - | - | - | - | 11 |
| Owusu-Akyaw et al., 2021 | III | 2 | 1 | 1 | 2 | 2 | 2 | 0 | 0 | - | - | - | - | 10 |
| Ramkumar et al., 2021a | III | 2 | 1 | 1 | 2 | 2 | 2 | 1 | 0 | - | - | - | - | 11 |
| Ramkumar et al., 2021b | III | 2 | 1 | 1 | 2 | 2 | 2 | 1 | 0 | - | - | - | - | 11 |
| LOE, level of evidence |  |  |  |  |  |  |  |  |  |  |  |  |  |  |

**Supplementary Table 7.** Detailed extracted baseline factor–outcome associations and separately identified post-treatment correlations.

| Study | Disease context | Procedure/analysis group | Factor/marker and coding | Outcome | Follow-up | Analysis/model | Effect estimate (95% CI) | Adjustment/covariates | QUIPS assessment ID and overall risk of bias |
| --- | --- | --- | --- | --- | --- | --- | --- | --- | --- |
| Saad Berreta et al., 2026 | Focal defect | Mixed MFx ± BMAC cohort | Weight (continuous) | IKDC MCID; IKDC PASS | Mean 7.1 y | Multivariable logistic regression | MCID: OR 0.98 (95% CI 0.96–0.99), P=.03 PASS: OR 0.98 (95% CI 0.97–0.99), P=.04 | AIC-selected multivariable models | Q01; High risk |
| Saad Berreta et al., 2026 | Focal defect | Mixed MFx ± BMAC cohort | Lower baseline PROM (continuous) | MCID: IKDC, KOOS ADL, Pain, Sport, Symptoms | Mean 7.1 y | Multivariable logistic regression | IKDC: OR 0.93 (0.90–0.95), P=.01 ADL: OR 0.75 (0.59–0.86), P<.01 Pain: OR 0.92 (0.85–0.98), P=.02 Sport: OR 0.94 (0.90–0.97), P<.01 Symptoms: OR 0.92 (0.87–0.97), P<.01 | AIC-selected multivariable models | Q01; High risk |
| Saad Berreta et al., 2026 | Focal defect | Mixed MFx ± BMAC cohort | Lesion size (continuous) | MCID: IKDC; KOOS Sport | Mean 7.1 y | Multivariable logistic regression | IKDC: OR 0.17 (0.02–0.86), P=.04 Sport: OR 0.09 (0.01–0.47), P=.01 | AIC-selected multivariable models | Q01; High risk |
| Saad Berreta et al., 2026 | Focal defect | Mixed MFx ± BMAC cohort | BMI (continuous) | MCID: KOOS Pain, Sport; PASS: KOOS ADL, Pain, Symptoms | Mean 7.1 y | Multivariable logistic regression | MCID Pain: OR 0.85 (0.71–0.98), P=.04 MCID Sport: OR 0.81 (0.68–0.95), P=.01 PASS ADL: OR 0.80 (0.64–0.94), P=.02 PASS Pain: OR 0.84 (0.70–0.97), P=.03 PASS Symptoms: OR 0.86 (0.73–0.98), P=.03 | AIC-selected multivariable models | Q01; High risk |
| Saad Berreta et al., 2026 | Focal defect | Mixed MFx ± BMAC cohort | Kellgren–Lawrence grade 0–1 vs 2–3 | PASS: IKDC, KOOS Pain, Symptoms | Mean 7.1 y | Multivariable logistic regression | IKDC: OR 4.12 (1.29–14.82), P=.02 Pain: OR 4.23 (1.30–15.32), P=.02 Symptoms: OR 5.46 (1.64–20.15), P<.01 | AIC-selected multivariable models | Q01; High risk |
| Saad Berreta et al., 2026 | Focal defect | Mixed MFx ± BMAC cohort | Age (continuous) | PASS: KOOS ADL, Symptoms | Mean 7.1 y | Multivariable logistic regression | ADL: OR 0.94 (0.89–0.99), P=.02 Symptoms: OR 0.95 (0.90–0.99), P=.02 | AIC-selected multivariable models | Q01; High risk |
| Saad Berreta et al., 2026 | Focal defect | Mixed MFx ± BMAC cohort | Male sex | KOOS Symptoms MCID | Mean 7.1 y | Multivariable logistic regression | OR 3.67 (95% CI 1.13–33.44), P=.04 | AIC-selected multivariable model | Q01; High risk |
| Saad Berreta et al., 2026 | Focal defect | MFx + BMAC vs MFx alone | BMAC augmentation | MCID and PASS across IKDC/KOOS | Mean 7.1 y | Unadjusted subgroup comparisons | No significant difference across outcomes; effect estimate/95% CI NR | Unadjusted; small BMAC subgroup | Q02; High risk |
| Koch et al., 2026 | Focal defect | Cell-based implantation without marrow stimulation | Male sex | Achievement of PASS across PROMs | ≥10 y | Multivariable binary logistic regression | OR 3.1 (95% CI 1.2–7.7), P=.016 | Adjusted for the variables shown in the model | Q03; High risk |
| Koch et al., 2026 | Focal defect | Cell-based implantation without marrow stimulation | BMI 20–29 kg/m² | Achievement of PASS across PROMs | ≥10 y | Multivariable binary logistic regression | OR 3.9 (95% CI 1.5–9.9), P=.004 | Adjusted for the variables shown in the model | Q03; High risk |
| Koch et al., 2026 | Focal defect | Cell-based implantation without marrow stimulation | Immature articular cartilage | Achievement of PASS across PROMs | ≥10 y | Multivariable binary logistic regression | OR 1.9 (95% CI 0.7–5.7), P=.229 | Adjusted for the variables shown in the model | Q03; High risk |
| Koch et al., 2026 | Focal defect | Cell-based implantation without marrow stimulation | >1 previous knee surgery | Achievement of PASS across PROMs | ≥10 y | Multivariable binary logistic regression | OR 0.5 (95% CI 0.1–1.6), P=.246 | Adjusted for the variables shown in the model | Q03; High risk |
| Kim et al., 2023 | OA-associated | Marrow stimulation + biologic adjunct | Lesion size: <3.5 (ref), 3.5–5.5, 5.5–7.5, ≥7.5 cm² | Unsatisfactory VAS/MOCART outcome | 12 mo | Multivariable logistic regression | OR 2.67 (0.23–31.07); 7.80 (0.65–93.81); 13.4 (2.49–215.36), respectively; overall P=.038 | Adjusted for age, sex, side, BMI, lesion size, and SVF cell count | Q04; High risk |
| Kim et al., 2023 | OA-associated | Marrow stimulation + biologic adjunct | SVF cells: ≥8.0×10⁷ (ref), 7.0–8.0×10⁷, <7.0×10⁷ | Unsatisfactory VAS/MOCART outcome | 12 mo | Multivariable logistic regression | OR 1.80 (0.43–7.53) and 7.20 (0.64–81.54), respectively; overall P=.021 | Adjusted for age, sex, side, BMI, lesion size, and SVF cell count | Q04; High risk |
| Kim et al., 2023 | OA-associated | Marrow stimulation + biologic adjunct | Age, sex, side, BMI, and lesion location | Unsatisfactory VAS/MOCART outcome | 12 mo | Multivariable logistic regression | No independent association; category-specific ORs reported for age, sex, side, and BMI; overall 95% CI not applicable | Mutually adjusted model | Q04; High risk |
| Seiferth et al., 2022 | Focal defect | Cell-based implantation without marrow stimulation | Previous knee surgery vs none | KOOS, VAS, reoperation, satisfaction | 6–36 mo | Propensity-score-matched group comparisons | Group means/proportions reported; common adjusted effect estimate and 95% CI NR | Matched on clinical and defect characteristics | Q05a; High risk |
| Seiferth et al., 2022 | Focal defect | Cell-based implantation without marrow stimulation | Previous cartilage treatment at index defect vs none | KOOS, VAS, reoperation, satisfaction | 6–36 mo | Propensity-score-matched group comparisons | Lower KOOS at 12–36 mo and lower satisfaction at 36 mo; common effect estimate and 95% CI NR | Matched on clinical and defect characteristics | Q05b; High risk |
| Seiferth et al., 2022 | Focal defect | Cell-based implantation without marrow stimulation | Type of previous cartilage treatment: BMS vs ACI | KOOS and change in KOOS | 6–36 mo | Matched subgroup comparison | No significant difference; effect estimate and 95% CI NR | Propensity-score-matched cohorts | Q05b; High risk |
| Wang et al., 2021 | Focal defect | Synthetic scaffold group only | Male sex; scaffold plugs; baseline SF-36 | SF-36 PCS | 2 y | Multivariable linear regression | Male: B 8.4, SE 3.6, P=.03 Plugs: B 3.5, SE 2.1, P=.11 Baseline: B 0.5, SE 0.1, P<.01 95% CI NR | Scaffold group; backward selection | Q06; Moderate risk |
| Wang et al., 2021 | Focal defect | Synthetic scaffold group only | Sex, BMI, previous MFx, location, etiology, baseline score | KOS-ADL | 2 y | Multivariable linear regression | Male: B −5.1, SE 2.5, P=.05 BMI: B −0.4, SE 0.2, P=.05 Previous MFx: B −12.6, SE 6.2, P=.06 LFC vs MFC: B 4.8, SE 2.9, P=.12 Trauma: B −8.0, SE 2.9, P=.01 Baseline: B 0.5, SE 0.1, P<.01 95% CI NR | Scaffold group; backward selection | Q06; Moderate risk |
| Wang et al., 2021 | Focal defect | Synthetic scaffold group only | Age, BMI, previous MFx, location, etiology, baseline score | IKDC | 2 y | Multivariable linear regression | Age: B −0.6, SE 0.2, P<.01 BMI: B −0.7, SE 0.2, P<.01 Previous MFx: B −31.4, SE 7.4, P<.01 LFC vs MFC: B 6.8, SE 3.6, P=.08 Trauma: B −10.9, SE 3.8, P=.01 Baseline: B 0.4, SE 0.1, P<.01 95% CI NR | Scaffold group; backward selection | Q06; Moderate risk |
| Wang et al., 2021 | Focal defect | Synthetic scaffold group only | Age, sex, BMI, location, etiology | Marx activity | 2 y | Multivariable linear regression | Age: B −0.2, SE 0.0, P<.01 Male: B 2.4, SE 1.1, P=.04 BMI: B −0.2, SE 0.1, P=.01 LFC vs MFC: B 2.4, SE 1.2, P=.07 Trauma: B 3.2, SE 1.6, P=.05 95% CI NR | Scaffold group; adjusted for baseline Marx score | Q06; Moderate risk |
| Chahla et al., 2020 | Focal defect | Isolated marrow stimulation | Lesion size (continuous and >2 cm²) | MCID achievement | 6 mo | Multivariable logistic regression | OR 0.41, P=.004; OR 0.89, P=.048; 95% CI NR | Candidate variables included age, BMI, sex, smoking, lesion size, and baseline KOOS | Q07; High risk |
| Chahla et al., 2020 | Focal defect | Isolated marrow stimulation | Age | MCID achievement | 24 mo | Multivariable logistic regression | OR 0.61, P=.036; 95% CI NR | Multivariable model | Q07; High risk |
| Chahla et al., 2020 | Focal defect | Isolated marrow stimulation | Male sex; baseline KOOS Symptoms; baseline KOOS Pain | PASS achievement | 6 mo | Multivariable logistic regression | OR 2.02, P=.016; OR 1.03, P=.049; OR 1.08, P=.040; 95% CI NR | Multivariable model | Q07; High risk |
| Chahla et al., 2020 | Focal defect | Isolated marrow stimulation | Male sex; age; baseline KOOS Pain | PASS achievement | 24 mo | Multivariable logistic regression | OR 2.70, P=.017; OR 0.86, P=.031; OR 1.10, P=.048; 95% CI NR | Multivariable model | Q07; High risk |
| Chahla et al., 2020 | Focal defect | Isolated marrow stimulation | Evaluated candidate variables | MCID and PASS achievement | 12 mo | Multivariable logistic regression | No independent predictor; effect estimates and 95% CI NR | Multivariable model | Q07; High risk |
| Solheim et al., 2018 | Focal defect | Pooled MFx + mosaicplasty cohort | Single lesion; normal surrounding cartilage; baseline Lysholm ≥50; symptoms <36 mo; no PF involvement; age ≤25; defect ≤3 cm² | Good/excellent outcome (Lysholm ≥80) | Median 14 y | Unadjusted dichotomized analyses | OR 4.041 (1.618–10.093); 2.774 (1.469–5.240); 2.711 (1.459–5.039); 2.452 (1.300–4.628); 2.274 (1.086–4.760); 2.230 (0.998–4.985); 2.204 (1.195–4.062), respectively | Unadjusted; excluded from procedure-specific synthesis | Q08; High risk |
| Solheim et al., 2018 | Focal defect | Pooled MFx + mosaicplasty cohort | Multiple lesions; baseline Lysholm <50; degenerative surrounding cartilage; symptoms ≥36 mo; partial meniscectomy; defect ≥4 cm² | Poor outcome (Lysholm <64 and/or knee replacement) | Median 14 y | Unadjusted dichotomized analyses | OR 2.840 (1.451–5.561); 2.478 (1.407–4.365); 2.305 (1.315–4.039); 2.020 (1.106–3.688); 1.903 (1.012–3.765); 1.768 (1.017–3.073), respectively | Unadjusted; excluded from procedure-specific synthesis | Q08; High risk |
| Solheim et al., 2018 | Focal defect | Pooled MFx + mosaicplasty cohort | Surgical technique, sex, trauma, side, meniscal lesion, PF involvement | Good or poor Lysholm outcome | Median 14 y | Unadjusted tests | No significant association; effect estimate/95% CI NR | Unadjusted; no factor-by-treatment interaction | Q08; High risk |
| Kim et al., 2015 | OA-associated | Cell-based implantation without marrow stimulation | BMI ≥27.5 vs <27.5 kg/m² | IKDC and Tegner | Mean 28.6 mo | Unadjusted subgroup comparison | Worse IKDC and Tegner; P=.022 and .005; effect estimate/95% CI NR | Unadjusted | Q09; High risk |
| Kim et al., 2015 | OA-associated | Cell-based implantation without marrow stimulation | Lesion size ≥5.7 vs <5.7 cm² | IKDC and Tegner | Mean 28.6 mo | Unadjusted subgroup comparison | Worse IKDC and Tegner; P=.012 and .024; effect estimate/95% CI NR | Unadjusted | Q09; High risk |
| Kim et al., 2015 | OA-associated | Cell-based implantation without marrow stimulation | Lesion size | ICRS repair grade | Second-look arthroscopy | Correlation/subgroup analysis | P=.001 and .048 in the two treatment groups; effect estimate/95% CI NR | Unadjusted | Q09; High risk |
| Kim et al., 2015 | OA-associated | Cell-based implantation without marrow stimulation | Age, sex, lesion location, subchondral cyst | IKDC, Tegner, ICRS grade | Mean 28.6 mo | Unadjusted subgroup comparisons | No significant association; effect estimate/95% CI NR | Unadjusted | Q09; High risk |
| Koh et al., 2014 | OA-associated | Cell-based implantation without marrow stimulation | BMI ≥27.5 vs <27.5 kg/m² | IKDC, Tegner, ICRS grade | Mean 26.5 mo | Unadjusted subgroup comparison | Worse outcomes; P=.014, .039, and .020; effect estimate/95% CI NR | Unadjusted | Q10; High risk |
| Koh et al., 2014 | OA-associated | Cell-based implantation without marrow stimulation | Lesion size ≥5.4 vs <5.4 cm² | IKDC, Tegner, ICRS grade | Mean 26.5 mo | Unadjusted subgroup comparison | Worse outcomes; P=.016, .011, and .041; effect estimate/95% CI NR | Unadjusted | Q10; High risk |
| Koh et al., 2014 | OA-associated | Cell-based implantation without marrow stimulation | Age, sex, lesion location, subchondral cyst | IKDC, Tegner, ICRS grade | Mean 26.5 mo | Unadjusted subgroup comparisons | No significant association; effect estimate/95% CI NR | Unadjusted | Q10; High risk |
| Vanlauwe et al., 2011 | Focal defect | CCI vs MFx interaction | Symptom duration <3 vs ≥3 y | Change in overall KOOS | 60 mo | Adjusted subgroup/treatment-interaction analysis | <3 y: between-treatment difference 10.69 (95% CI 1.30–20.07), P=.026 ≥3 y: CCI 13.09±4.78 vs MFx 17.02±4.50, P=.554 Exact interaction estimate/P NR | Adjusted for baseline overall KOOS, age, associated lesions, and lesion location | Q11a; Moderate risk |
| Vanlauwe et al., 2011 | Focal defect | CCI treatment group | Female vs male sex | Treatment failure/reintervention | 60 mo | Unadjusted Fisher exact test | RR 4.21 (95% CI 1.03–17.57), P=.007 | Unadjusted treatment-specific association | Q11b; High risk |
| Vanlauwe et al., 2011 | Focal defect | MFx treatment group | Female vs male sex | Treatment failure/reintervention | 60 mo | Unadjusted Fisher exact test | RR 4.78 (95% CI 1.49–15.62), P=.010 | Unadjusted treatment-specific association | Q11b; High risk |
| Vanlauwe et al., 2011 | Focal defect | CCI vs MFx comparison | Age <35 vs ≥35 y | Change in overall KOOS | 60 mo | Adjusted subgroup comparisons | Treatment contrasts: P=.262 and P=.465; effect estimates/95% CI for interaction NR | Adjusted efficacy analysis; no procedure-specific age estimate | Q11c; High risk |
| Asik et al., 2008 | Focal defect | Isolated marrow stimulation | Age <35 vs ≥35 y | Lysholm, Tegner, Oxford scores | Mean 68 mo | Unadjusted subgroup/Spearman analyses | Group means: 36.2 vs 24.3; 2.6 vs 2.1; 21.7 vs 16.5 r=0.623, 0.612, 0.615; all P<.001; 95% CI NR | Unadjusted | Q12; High risk |
| Asik et al., 2008 | Focal defect | Isolated marrow stimulation | Defect <2 vs ≥2 cm² | Lysholm, Tegner, Oxford scores | Mean 68 mo | Unadjusted subgroup/Spearman analyses | Group means: 37.4 vs 26.9; 2.8 vs 2.0; 22.2 vs 15.8 r=0.538, 0.562, 0.576; all P<.001; 95% CI NR | Unadjusted | Q12; High risk |
| Asik et al., 2008 | Focal defect | Isolated marrow stimulation | Non-weight-bearing vs weight-bearing location | Lysholm, Tegner, Oxford scores | Mean 68 mo | Unadjusted subgroup/Spearman analyses | Group means: 37.3 vs 26.8; 2.6 vs 2.2; 23.2 vs 16.2 r=0.658, 0.629, 0.618; all P<.001; 95% CI NR | Unadjusted | Q12; High risk |
| Asik et al., 2008 | Focal defect | Isolated marrow stimulation | BMI <25 vs ≥25 kg/m² | Lysholm, Tegner, Oxford scores | Mean 68 mo | Unadjusted subgroup/Spearman analyses | Group means: 38.2 vs 26.2; 2.8 vs 2.0; 22.8 vs 16.3 r=0.547, 0.589, 0.572; all P<.001; 95% CI NR | Unadjusted | Q12; High risk |
| Asik et al., 2008 | Focal defect | Isolated marrow stimulation | Symptoms <12 vs ≥12 mo | Functional scores | Mean 68 mo | Unadjusted comparison | No significant association at final follow-up; effect estimate/95% CI NR | Unadjusted | Q12; High risk |
| Kreuz et al., 2006a | Focal defect | Isolated marrow stimulation | Lesion location | ICRS, Cincinnati, MRI defect fill | 36 mo | Unadjusted group comparisons | Femoral condyle group had better clinical scores and fill than other locations; P<.02; 95% CI NR | Unadjusted | Q13a; High risk |
| Kreuz et al., 2006a | Focal defect | Isolated marrow stimulation | Age <40 vs ≥40 y | ICRS score | 36 mo | Unadjusted subgroup comparison | Younger patients had better outcome, P=.008; older patients deteriorated, P=.005; effect estimate/95% CI NR | Unadjusted | Q13a; High risk |
| Kreuz et al., 2006a | Focal defect | Isolated marrow stimulation | Postoperative MRI defect fill (post-treatment correlate; not a baseline prognostic factor) | ICRS score | 36 mo | Pearson correlation | r=0.84, P<.01; 95% CI NR | Unadjusted; postoperative correlate | Q13b; High risk |
| Kreuz et al., 2006b | Focal defect | Isolated marrow stimulation | Age ≤40 vs >40 y | ICRS and Cincinnati scores | 6–36 mo | Unadjusted subgroup/repeated comparisons | Better scores in younger patients, P<.01; effect estimate/95% CI NR | Unadjusted; overlapping report of the same cohort as Kreuz 2006a; not counted independently in the synthesis | Q14a; High risk |
| Kreuz et al., 2006b | Focal defect | Isolated marrow stimulation | Lesion location | ICRS/Cincinnati and MRI outcomes | 36 mo | Unadjusted group comparisons | Femoral condyle location had best outcomes, P<.02; effect estimate/95% CI NR | Unadjusted; overlapping report of the same cohort as Kreuz 2006a; not counted independently in the synthesis | Q14a; High risk |
| Kreuz et al., 2006b | Focal defect | Isolated marrow stimulation | Postoperative MRI defect fill (post-treatment correlate; not a baseline prognostic factor) | ICRS score | 36 mo | Correlation | r=0.84, P<.01; 95% CI NR | Unadjusted; postoperative correlate; duplicates Kreuz 2006a and was not counted independently in the synthesis | Q14b; High risk |
| Kreuz et al., 2006a/b | Focal defect | Isolated marrow stimulation | Lesion size, BMI, defect grade, symptom duration | Clinical scores | 36 mo | Unadjusted analyses | No significant association; effect estimate/95% CI NR | Confirmed overlapping reports of the same cohort; duplicate estimates counted once | Q13a/Q14a; High risk |
| Steadman et al., 2003 | Focal defect | Isolated marrow stimulation | Age, chronicity, location, lesion size | Change in Lysholm score | Mean 11.3 y | Backward multivariable linear regression | Age: standardized B −0.299, P=.011 Chronicity: B −0.084, P=.466 Location: B −0.226, P=.066 Size: B −0.146, P=.225 95% CI NR | Mutually adjusted model; model R²=.193 | Q15; High risk |
| Philippe et al., 2026 | Focal defect | Cell-based implantation without marrow stimulation | Initial chondrocyte yield (>10^5 vs lower) | KOOS Symptoms/Pain/ADL/Sport change | Mean 4.2 y | Multiple linear regression | β 63.56, P<.01; β 88.83, P<.001; β 107.84, P<.001; β 92.30, P<.05; 95% CIs NR | Multiple model; covariate list incompletely reported in main article | Q16; High risk |
| Philippe et al., 2026 | Focal defect | Cell-based implantation without marrow stimulation | Longer preimplantation population-doubling time | IKDC/KOOS Pain/KOOS ADL change | Mean 4.2 y | Multiple linear regression | β −26.12, P<.01; β −23.09, P<.05; β −21.71, P<.05; 95% CIs NR | Multiple model; covariate list incompletely reported in main article | Q16; High risk |
| Tierney et al., 2025 | Focal defect | Cell-based implantation without marrow stimulation | Age (per year) | 1-year Lysholm | 1 y | Multilevel longitudinal model | B −0.21 (95% CI −0.42 to −0.00), P=.047 | Final model variables mutually adjusted | Q17; High risk |
| Tierney et al., 2025 | Focal defect | Cell-based implantation without marrow stimulation | Implanted-cell number (per natural-log unit; 2.7-fold increase) | 1-year Lysholm | 1 y | Multilevel longitudinal model | B −5.29 (95% CI −10.56 to −0.02), P=.049 | Final model variables mutually adjusted | Q17; High risk |
| Tierney et al., 2025 | Focal defect | Cell-based implantation without marrow stimulation | Baseline Lysholm (per point) | 1-year Lysholm | 1 y | Multilevel longitudinal model | B 0.47 (95% CI 0.34–0.61), P<.001 | Final model variables mutually adjusted | Q17; High risk |
| Tierney et al., 2025 | Focal defect | Cell-based implantation without marrow stimulation | Lateral vs medial femoral-condyle defect | 1-year Lysholm | 1 y | Multilevel longitudinal model | B 6.27 (95% CI 0.31–12.23), P=.039 | Final model variables mutually adjusted | Q17; High risk |
| Tierney et al., 2025 | Focal defect | Cell-based implantation without marrow stimulation | ICRS grade 4 vs grade 3 × time | Longitudinal Lysholm slope | 1–23 y | Multilevel longitudinal model | B −0.96 points/y (95% CI −1.79 to −0.14), P=.023 | Final model variables mutually adjusted | Q17; High risk |
| Cook et al., 2025 | Focal defect | Osteochondral procedure | Concurrent ligament reconstruction | Initial OCAT treatment failure | Mean 79 mo | Unadjusted comparison/reported OR | OR 2.5, P=.017; 95% CI NR | No multivariable adjustment | Q18; High risk |
| Cook et al., 2025 | Focal defect | Osteochondral procedure | Staged or concurrent ipsilateral osteotomy | Initial OCAT treatment failure | Mean 79 mo | Unadjusted comparison/reported OR | OR 2.4, P=.019; 95% CI NR | No multivariable adjustment | Q18; High risk |
| Cook et al., 2025 | Focal defect | Osteochondral procedure | Tibiofemoral bipolar plus other-surface OCAT | Initial OCAT treatment failure | Mean 79 mo | Unadjusted comparison/reported OR | OR 8.9, P<.0001; 95% CI NR | No multivariable adjustment | Q18; High risk |
| Yazdi et al., 2025 | Focal defect | Osteochondral procedure | Male vs female sex | IKDC MCID; IKDC SCB | ≥5 y | Multivariable logistic regression | MCID OR 0.234 (95% CI 0.086–0.636), P=.004; SCB OR 0.433 (0.205–0.917), P=.028 | Outcome-specific candidate-variable models | Q19; High risk |
| Yazdi et al., 2025 | Focal defect | Osteochondral procedure | Number of previous surgeries (per procedure) | IKDC MCID | ≥5 y | Multivariable logistic regression | OR 0.610 (95% CI 0.429–0.868), P=.006 | Outcome-specific candidate-variable model | Q19; High risk |
| Yazdi et al., 2025 | Focal defect | Osteochondral procedure | Workers’ compensation | KOOS Pain MCID; KOOS QOL MCID; KOOS Pain/Sport PASS; KOOS Sport/QOL SCB | ≥5 y | Multivariable logistic regression | ORs 0.026, 0.081, 0.202, 0.188, 0.165 and 0.171; all P≤.017 (CIs reported in source Table 6–8) | Outcome-specific candidate-variable models | Q19; High risk |
| Yazdi et al., 2025 | Focal defect | Osteochondral procedure | Medial/lateral femoral or trochlear defect location | Selected KOOS MCID/PASS outcomes | ≥5 y | Multivariable logistic regression | Lower odds for several outcome-specific thresholds; adjusted OR range 0.069–0.406 for significant estimates | Outcome-specific candidate-variable models; reference was absence of the named location | Q19; High risk |
| Wang et al., 2025 | Focal/mixed osteochondral diagnoses | Osteochondral procedure | Age ≥30 vs <30 y | OCA failure | Mean 8.2 y | Multivariable logistic regression | OR 2.13 (95% CI 1.14–4.00), P=.018 | Age, BMI, diagnosis and total graft size in final model | Q20; Moderate risk |
| Wang et al., 2025 | Focal/mixed osteochondral diagnoses | Osteochondral procedure | BMI ≥30 vs <30 kg/m² | OCA failure | Mean 8.2 y | Multivariable logistic regression | OR 2.24 (95% CI 1.21–4.16), P=.011 | Age, BMI, diagnosis and total graft size in final model | Q20; Moderate risk |
| Wang et al., 2025 | Focal/mixed osteochondral diagnoses | Osteochondral procedure | Diagnosis vs osteochondritis dissecans | OCA failure | Mean 8.2 y | Multivariable logistic regression | Degenerative lesion OR 2.47 (1.15–5.32), P=.021; AVN OR 4.57 (1.76–11.81), P=.002; OA OR 3.11 (1.19–8.14), P=.021 | Age, BMI, diagnosis and total graft size in final model | Q20; Moderate risk |
| Wang et al., 2025 | Focal/mixed osteochondral diagnoses | Osteochondral procedure | Total graft area >8 vs ≤8 cm² | OCA failure | Mean 8.2 y | Multivariable logistic regression | OR 2.12 (95% CI 1.18–3.84), P=.013 | Age, BMI, diagnosis and total graft size in final model | Q20; Moderate risk |
| Ebert et al., 2025 | Focal defect | Cell-based implantation without marrow stimulation | Defect size (cm²) | 10-year MRI composite score | 10 y | Multivariable linear regression | B −0.07 (95% CI −0.13 to −0.01); standardized β −0.247; P=.015 | Only predictor retained in final model | Q21; Moderate risk |
| Ebert et al., 2025 | Focal defect | Cell-based implantation without marrow stimulation | Prior knee procedures (number) | 10-year tissue infill | 10 y | Multivariable ordinal logistic regression as reported | B −0.66 (95% CI −1.17 to −0.14), P=.012 | Adjusted with defect size and baseline KOOS Sport/Rec | Q21; Moderate risk |
| Ebert et al., 2025 | Focal defect | Cell-based implantation without marrow stimulation | Defect size (cm²) | 10-year tissue infill | 10 y | Multivariable ordinal logistic regression as reported | B −0.34 (95% CI −0.60 to −0.08), P=.012 | Adjusted with prior procedures and baseline KOOS Sport/Rec | Q21; Moderate risk |
| Weishorn et al., 2024 | Focal defect | Cell-based implantation without marrow stimulation | BMI (kg/m²) | Final KOOS | Mean 8.1 y | Multivariable linear regression | B −0.8, P=.033; 95% CI NR | Adjusted for age, MOCART, previous surgery and cell count; n=59 | Q22; High risk |
| Weishorn et al., 2024 | Focal defect | Cell-based implantation without marrow stimulation | MOCART 2.0 score | Final KOOS | Mean 8.1 y | Multivariable linear regression | B 0.2, P=.015; 95% CI NR | Adjusted for age, BMI, previous surgery and cell count; postoperative imaging correlation | Q22; High risk |
| Weishorn et al., 2024 | Focal defect | Cell-based implantation without marrow stimulation | Number of previous knee surgeries | Final KOOS | Mean 8.1 y | Multivariable linear regression | B −5.6, P=.009; 95% CI NR | Adjusted for age, BMI, MOCART and cell count; n=59 | Q22; High risk |
| Sato et al., 2024 | Focal defect | Mixed cartilage-restoration cohort | Maximum preoperative WORMS | Lysholm/PROMIS PF-CAT/VAS pain | Mean 3.6 y | Spearman correlation | r −0.52 (95% CI −0.72 to −0.21); r −0.39 (−0.64 to −0.068); r 0.47 (0.16–0.69) | Unadjusted mixed-procedure analysis | Q23; High risk |
| Sato et al., 2024 | Focal defect | Mixed cartilage-restoration cohort | Preoperative meniscus/cartilage/BMELL WORMS subscales | Selected postoperative PROMs | Mean 3.6 y | Spearman correlation | Meniscus–Lysholm r −0.36; cartilage–Lysholm r −0.37; cartilage–KOOS ADL r −0.40; cartilage–KOOS Sport r −0.36; BMELL–VAS pain r 0.36 (all source CIs exclude 0) | Unadjusted mixed-procedure analysis | Q23; High risk |
| Bumberger et al., 2023 | Focal defect | Mixed cartilage-restoration cohort | Bipolar vs unipolar lesion | Change in overall KOOS | 6, 12, 24 and 36 mo | Propensity-score-matched comparison | Mean change lower for bipolar lesions at all horizons: between-group P=.020, .007, .003 and .024 | Matched for age, sex, BMI, previous surgery, lesion location and size | Q24; High risk |
| Bumberger et al., 2023 | Focal defect | Mixed cartilage-restoration cohort | Bipolar vs unipolar lesion | 24-month KOOS MCID achievement | 24 mo | Propensity-score-matched comparison | 45.5% vs 64.8%, P=.029; effect estimate/95% CI NR | Matched baseline groups; follow-up attrition remained | Q24; High risk |
| Bumberger et al., 2023 | Focal defect | Mixed cartilage-restoration cohort | Bipolar vs unipolar lesion | Revision/failure | Up to 60 mo | Matched comparison/Kaplan–Meier | Failure 8.2% vs 3.9%, P=.256; mean survival P=.195 | Matched baseline groups; imprecise event comparison | Q24; High risk |
| Byrne et al., 2023 | Focal defect | Osteochondral procedure | Age 20–29 vs 50–59 y | Salvage surgery after OCA | 5 y | Multivariable logistic regression | aOR 0.49 (95% CI 0.24–0.99), P=.046 | Adjusted claims model | Q25; Moderate risk |
| Byrne et al., 2023 | Focal defect | Osteochondral procedure | Prior or concomitant bony realignment | Salvage surgery after OCA | 5 y | Multivariable logistic regression | aOR 0.24 (95% CI 0.04–0.76), P=.046 | Adjusted claims model | Q25; Moderate risk |
| Kim et al., 2020 | Osteoarthritis-associated lesion | Cell-based implantation without marrow stimulation | Age ≥70 vs <50 y | MSC-implantation failure | 5–9 y | Multivariable Cox regression | HR 6.726 (95% CI 2.346–19.283); age overall P=.002 | All listed baseline factors in Cox model | Q26; Moderate risk |
| Kim et al., 2020 | Osteoarthritis-associated lesion | Cell-based implantation without marrow stimulation | Bipolar kissing lesion present vs absent | MSC-implantation failure | 5–9 y | Multivariable Cox regression | HR 2.626 (95% CI 1.222–5.646), P=.013 | All listed baseline factors in Cox model | Q26; Moderate risk |
| Kim et al., 2020 | Osteoarthritis-associated lesion | Cell-based implantation without marrow stimulation | Implanted MSC number categories | MSC-implantation failure | 5–9 y | Multivariable Cox regression | Global P<.001; category HRs 0.856, 0.470 and 0.144 vs <5×10^6, with all reported 95% CIs crossing 1 | All listed baseline factors in Cox model | Q26; Moderate risk |
| Merkely et al., 2021 | Focal defect | ACI and OCA (procedure-specific analyses) | Defect size (per cm²), ACI cohort | ACI graft failure | Procedure-specific follow-up | Multivariable logistic regression | OR 1.07 (95% CI 1.01–1.14), P=.035 | Adjusted for age, sex, hypertension and smoking | Q27; High risk |
| Merkely et al., 2021 | Focal defect | ACI and OCA (procedure-specific analyses) | Hypertension, ACI cohort | ACI graft failure | Procedure-specific follow-up | Multivariable logistic regression | OR 3.73 (95% CI 1.20–11.6), P=.023 | Adjusted for age, sex, defect size and smoking | Q27; High risk |
| Merkely et al., 2021 | Focal defect | ACI and OCA (procedure-specific analyses) | Female sex, ACI cohort | ACI graft failure | Procedure-specific follow-up | Multivariable logistic regression | Published OR 1.02 (95% CI 1.01–3.64), P=.048; coefficient 0.648 is internally inconsistent with the printed OR | Adjusted for age, defect size, hypertension and smoking; source-report inconsistency flagged | Q27; High risk |
| Wright-Chisem et al., 2021 | Focal defect | Osteochondral procedure | Number of self-reported drug allergies | KOS-ADL/IKDC/Marx/VAS MCID | ≥2 y | Multivariable logistic regression | IKDC OR 0.78, P=.27; Marx 0.91, P=.75; VAS pain 1.3, P=.38; health state 1.2, P=.71; KOS-ADL 2.0, P=.056; 95% CIs NR | Adjusted for age, BMI and sex | Q28; Moderate risk |
| Gillinov et al., 2022 | Focal defect | Cell-based implantation without marrow stimulation | Female vs male sex | Revision after ACI | 5 y | Multivariable logistic regression | OR 2.58 (95% CI 1.22–5.45), P=.013 | Adjusted for age, BMI, ECI and bony realignment | Q29; Moderate risk |
| Gillinov et al., 2022 | Focal defect | Cell-based implantation without marrow stimulation | BMI ≥35 vs 20–24.9 kg/m² | Revision after ACI | 5 y | Multivariable logistic regression | OR 2.24 (95% CI 1.01–4.94), P=.047 | Adjusted for age, sex, ECI and bony realignment | Q29; Moderate risk |
| Owusu-Akyaw et al., 2021 | Focal defect | Osteochondral procedure | Preoperative SF-36 PCS (per point) | IKDC MCID achievement | 2 y | Multivariable logistic regression | OR 0.92 (95% CI 0.85–0.99), P=.02 | Adjusted for age, defect size and MCS in final model | Q30; High risk |
| Owusu-Akyaw et al., 2021 | Focal defect | Osteochondral procedure | Defect diameter >2 vs <2 cm | IKDC MCID achievement | 2 y | Multivariable logistic regression | OR 2.64 (95% CI 1.00–6.97), P=.0492 | Adjusted for age, PCS and MCS in final model | Q30; High risk |
| Ramkumar et al., 2021a | Focal defect | Osteochondral procedure | Mental health, physical/knee function, activity demand, pain and BMI | Failure to achieve MCID/SCB | 1 and 2 y | Machine-learning models with SHAP feature ranking | Top-model AUC 0.72–0.94; factor-specific effect estimates/95% CIs NR | Multiple algorithms; feature ranking is not a conventional adjusted association | Q31; High risk |
| Ramkumar et al., 2021b | Focal defect | Osteochondral procedure | BMI, malalignment, osseous edema, ACL/meniscal injury, defect size and OCA plug number | Failure to achieve functional MCID/SCB | 2 y | Machine-learning models with SHAP feature ranking | Functional-model AUC 0.84–0.90; factor-specific effect estimates/95% CIs NR | Same HSS cohort as record 333; imaging-focused feature ranking | Q32; High risk |
| ACI, autologous chondrocyte implantation; ADL, activities of daily living; AIC, Akaike information criterion; B, regression coefficient; BMAC, bone marrow aspirate concentrate; BMI, body mass index; CCI, characterized chondrocyte implantation; CI, confidence interval; ICRS, International Cartilage Repair Society; IKDC, International Knee Documentation Committee; KOOS, Knee injury and Osteoarthritis Outcome Score; LFC, lateral femoral condyle; MCID, minimal clinically important difference; MFC, medial femoral condyle; MFx, microfracture; MOCART, Magnetic Resonance Observation of Cartilage Repair Tissue; NR, not reported; OA, osteoarthritis; OR, odds ratio; PASS, patient acceptable symptom state; PF, patellofemoral; PROM, patient-reported outcome measure; RR, risk ratio; SE, standard error; SVF, stromal vascular fraction; VAS, visual analog scale. Effect estimates and confidence intervals are reproduced as reported in the primary studies; missing estimates were not inferred. QUIPS assessments were assigned at the study-analysis level; estimates from the same analysis/model share an assessment ID. Overall risk of bias was classified as high when at least one domain was high, moderate when at least one domain was moderate and no domain was high, and low only when all six domains were low. Domain judgments were not summed. Detailed domain-level judgments and supporting rationales are provided in Supplementary Table 8. Two report-level entries for postoperative MRI defect fill were retained solely to document post-treatment correlations reported from the same overlapping Kreuz cohort; they were not classified or synthesized as baseline prognostic factors. | | | | | | | | | |

**Supplementary Table 8.** QUIPS domain-level risk-of-bias judgments and supporting rationales for the included prognostic-factor analyses.

| QUIPS ID | Study/analysis unit | D1 | D2 | D3 | D4 | D5 | D6 | Overall risk of bias | Supporting rationale |
| --- | --- | --- | --- | --- | --- | --- | --- | --- | --- |
| Q01 | Saad Berreta et al., 2026: multivariable MCID/PASS models | Moderate | High | Low | Moderate | Moderate | High | High | D1: Single-center retrospective analysis with defined eligibility, but inclusion required complete long-term PROMs. D2: 70/103 eligible patients were analyzed; patients with incomplete final PROMs or subsequent ipsilateral surgery were excluded, without an attrition comparison. D3: Baseline factors were obtained from records, radiographs, and operative documentation. D4: Validated PROMs were used, but MCID/PASS thresholds were derived within the cohort. D5: Multivariable models were used, although AIC selection did not ensure inclusion of all core confounders. D6: The small sample supported many outcome-specific models and candidate predictors, creating overfitting and multiplicity concerns. |
| Q02 | Saad Berreta et al., 2026: BMAC augmentation subgroup comparison | Moderate | High | Low | Moderate | High | High | High | D1-D4: The same cohort limitations as Q01 apply. D5: The BMAC comparison was unadjusted. D6: Only 10 BMAC-treated patients were compared with 60 nonaugmented patients, and no effect estimate or confidence interval was reported. |
| Q03 | Koch et al., 2026: long-term PASS logistic model | Low | High | Low | Moderate | High | Moderate | High | D1: A consecutive cohort and eligibility criteria were reported. D2: Patients converted to knee replacement or withdrawing consent were excluded, along with three patients lost to follow-up, producing outcome-dependent attrition. D3: Prognostic factors were clearly defined. D4: Validated PROMs and an anchor question were used, but PASS thresholds were derived within the cohort. D5: The model included only sex, BMI, cartilage maturity, and previous surgery and omitted important lesion, baseline-score, and concomitant-treatment factors. D6: Estimates and CIs were reported, but the power calculation addressed ROC performance rather than the prognostic model. |
| Q04 | Kim et al., 2023: multivariable unsatisfactory-outcome model | Moderate | High | Low | High | Moderate | High | High | D1: Consecutive eligibility was described. D2: Only 43/62 qualified patients completed assessment; 14 dropped out and five were lost without comparison of completers and noncompleters. D3: Lesion size and SVF cell count were measured using reported clinical/laboratory procedures. D4: The composite unsatisfactory outcome used sample-derived mean cutoffs for VAS and MOCART. D5: Several patient factors were adjusted, but baseline outcome and disease-severity factors were not fully addressed. D6: Six predictors with multiple categories were modeled in 43 patients, with sparse cells and very wide CIs indicating substantial overfitting risk. |
| Q05a | Seiferth et al., 2022: previous knee surgery propensity-matched comparison | Moderate | High | Moderate | Moderate | Moderate | High | High | D1: The nationwide registry was well described, but participation required consent/email access and this analysis selected ACI recipients with intact menisci. D2: Outcome data were substantially incomplete (e.g., 730 matched patients with previous surgery but 482 baseline and 211 36-month KOOS observations), without missing-data modeling. D3: Previous surgery was recorded prospectively but remained a broad exposure category. D4: KOOS/VAS were validated; satisfaction and reintervention were more practice-dependent. D5: Extensive propensity matching reduced measured confounding, but residual confounding and baseline KOOS imbalance remained. D6: Repeated complete-case comparisons lacked a common effect estimate/CI and multiplicity correction. |
| Q05b | Seiferth et al., 2022: previous defect treatment and BMS-vs-ACI comparisons | Moderate | High | Moderate | Moderate | Moderate | High | High | D1-D4: Registry selection, substantial missing follow-up data, broad prior-treatment classification, and mixed outcome types were as described for Q05a. D5: Propensity matching addressed many measured variables, but treatment-history subgroups remained susceptible to residual confounding. D6: Multiple time-point and subgroup comparisons were reported without a common adjusted estimate, CI, or multiplicity correction. |
| Q06 | Wang et al., 2021: scaffold-group 2-year multivariable models | Moderate | Moderate | Low | Low | Moderate | Moderate | Moderate | D1: A prospective registry and eligibility criteria were reported, but the full eligible/participating pool was not described. D2: The 2-year response rate in the scaffold group was 54/66; later follow-up was more incomplete. D3: Baseline and operative predictors were clearly recorded. D4: Validated PROMs were collected by an independent observer. D5: Candidate confounders and baseline scores were considered, but backward selection produced model-specific omissions. D6: The analysis used a modest sample for several outcome-specific stepwise models and reported B, SE, and P values without 95% CIs. |
| Q07 | Chahla et al., 2020: MCID/PASS prognostic models | High | Moderate | Low | Moderate | Moderate | High | High | D1: Only 206/521 queried patients entered the analysis; 288 lacked baseline PROMs and were excluded without comparison. D2: A further 27 patients lacked minimum 2-year follow-up. D3: Candidate factors were clinically defined. D4: Validated PROMs were used, but thresholds were derived in the cohort and achievement of any included measure formed a composite outcome. D5: Multivariable models were fitted, but variables were screened by univariable significance and concomitant procedures were unavailable in more than 30%. D6: Numerous outcomes/time points were modeled without reported CIs, multiplicity control, or model-event adequacy. |
| Q08 | Solheim et al., 2018: pooled unadjusted determinant analyses | Moderate | Moderate | Moderate | Moderate | High | High | High | D1: Eligibility was described, but microfracture and mosaicplasty were selected by surgeon/patient preference and pooled. D2: All patients had at least one follow-up, but follow-up ranged from 1 to 18 years and the latest observation was used. D3: Several predictors were dichotomized and surrounding-cartilage status was a subjective surgeon assessment. D4: Lysholm was validated, but good/poor thresholds and a poor-outcome composite were imposed. D5: All prognostic analyses were unadjusted and treatment groups were pooled. D6: Numerous dichotomized univariable tests were performed without multivariable control. |
| Q09 | Kim et al., 2015: unadjusted prognostic comparisons/correlations | High | High | Low | Moderate | High | High | High | D1: Only 56/93 eligible knees underwent voluntary second-look arthroscopy, and much of one group overlapped a prior report. D2: Nonparticipation in second-look assessment was substantial. D3: Demographic and lesion factors were clinically documented. D4: Clinical scores were validated, but ICRS repair grade was assigned by a single senior surgeon without reported blinding. D5: Reported prognostic comparisons were unadjusted. D6: Small subgroups, median-derived cutoffs, multiple tests, and absent effect estimates/CIs created major analysis/reporting concerns. |
| Q10 | Koh et al., 2014: unadjusted prognostic comparisons | High | Moderate | Low | Moderate | High | High | High | D1: The source cohort was consecutive, but prognostic assessment relied mainly on the 37/60 knees undergoing voluntary second-look arthroscopy. D2: Second-look data were available for 62%, although clinical characteristics of participants and nonparticipants were compared. D3: Patient and lesion factors were defined. D4: IKDC/Tegner were validated; arthroscopic ICRS grading was subjective. D5: Prognostic comparisons were unadjusted. D6: The small sample, median cutoffs, multiple comparisons, and lack of effect estimates/CIs produced high analysis/reporting risk. |
| Q11a | Vanlauwe et al., 2011: symptom-duration treatment-effect analysis | Low | Moderate | Moderate | Low | Moderate | Moderate | Moderate | D1: The multicenter randomized cohort and eligibility criteria were clearly described. D2: Extension-phase participation and nonfailure missing data were incompletely characterized; LOCF was used only after documented failure. D3: Symptom duration may be recall-dependent and was dichotomized at 3 years. D4: KOOS was validated and assessments preceded the surgeon visit. D5: Analyses adjusted for baseline KOOS, age, associated lesions, and location, although residual subgroup confounding remained. D6: This was a planned exploratory subgroup analysis; multiple domains were examined and the exact interaction estimate and P value were not reported. |
| Q11b | Vanlauwe et al., 2011: sex-specific failure associations | Low | Moderate | Low | Moderate | High | High | High | D1-D3: The randomized cohort was well defined, with some long-term missing-data uncertainty; sex was measured reliably. D4: Reintervention was clinically defined but could reflect patient/surgeon decision making. D5: Sex-failure associations were unadjusted despite imbalances in symptom duration and baseline pain. D6: Only 17 failures were available and small treatment-specific Fisher tests were reported without multivariable verification. |
| Q11c | Vanlauwe et al., 2011: age subgroup treatment comparisons | Low | Moderate | Low | Low | High | High | High | D1-D4: The randomized cohort, long-term missing-data limitations, age measurement, and validated KOOS outcome were acceptable overall. D5: The reported statistics were treatment contrasts within age strata rather than procedure-specific age-outcome associations. D6: No age-by-treatment interaction estimate/CI was reported, and the analysis was exploratory. |
| Q12 | Asik et al., 2008: unadjusted prognostic analyses | High | High | Moderate | Low | High | High | High | D1: From 246 treated patients, 30 who underwent secondary surgery after the index procedure and 98 with other pathology were excluded, leaving a highly selected cohort of 90. D2: Twenty-eight were lost and outcome-related secondary-surgery exclusions could remove poor outcomes. D3: Predictors were dichotomized, with limited measurement detail for lesion location. D4: Lysholm, Tegner, and Oxford scores were established measures. D5: Analyses were unadjusted. D6: Multiple subgroup/correlation tests were reported without adjusted effects or CIs. |
| Q13a | Kreuz et al., 2006a: baseline prognostic analyses | Moderate | Low | Moderate | Moderate | High | High | High | D1: Seventy of 85 treated patients met reported eligibility criteria, but recruitment/participation details were limited. D2: The 70 patients appeared to complete the 36-month assessment. D3: Age and lesion characteristics were documented, but subgroup cutoffs and location groups were used. D4: Clinical scores were standardized, although examiner blinding was not reported. D5: Analyses were unadjusted. D6: Multiple group/time-point comparisons lacked adjusted estimates and CIs; this cohort likely overlaps Q14a. |
| Q13b | Kreuz et al., 2006a: postoperative MRI-fill correlation | Moderate | Low | High | Moderate | High | High | High | D1-D2: The same cohort and follow-up as Q13a were used. D3: MRI defect fill was measured after surgery and is an intermediate marker rather than a baseline prognostic factor. D4: The clinical score was standardized, but assessor blinding was not reported. D5: The correlation was unadjusted. D6: A single correlation without confounder control or CI was reported; the cohort likely overlaps Q14b. |
| Q14a | Kreuz et al., 2006b: baseline prognostic analyses | Moderate | Low | Moderate | Moderate | High | High | High | D1: The same 85 treated/70 included patients, period, and eligibility pattern as the companion Kreuz report were described. D2: The 70 patients appeared to complete 36 months. D3-D4: Predictor groups and standardized clinical/MRI outcomes were reported, but clinical assessor blinding was unclear. D5: Analyses were unadjusted. D6: Repeated subgroup/time-point testing lacked adjusted estimates/CIs and substantially overlaps Q13a, precluding independent evidence counting. |
| Q14b | Kreuz et al., 2006b: postoperative MRI-fill correlation | Moderate | Low | High | Moderate | High | High | High | D1-D2: The same likely overlapping Kreuz cohort was used. D3: MRI defect fill was a postoperative intermediate marker, not a baseline prognostic factor. D4: MRI was read by two radiologists and clinical scores were standardized, but independence/blinding was incompletely reported. D5: The correlation was unadjusted. D6: No adjusted estimate/CI was provided and the result duplicates the correlation in Q13b. |
| Q15 | Steadman et al., 2003: multivariable Lysholm-change model | Moderate | High | Moderate | Low | Moderate | Moderate | High | D1: A consecutive surgical cohort was described, with 72/302 meeting restrictive but explicit focal-defect criteria. D2: Two patients lost to follow-up and two treatment failures were deleted from analysis. D3: Age, chronicity, location, and size were recorded, with limited measurement detail. D4: Change in validated Lysholm score was used. D5: The four factors were mutually adjusted, but other core confounders were not addressed. D6: Backward selection was used in 71 knees; B, P, and R-squared were reported without CIs. |
| Q16 | Philippe et al., 2026: ACI manufacturing-factor multiple regression | Moderate | High | Moderate | Low | High | High | High | Small complete-case models, incomplete covariate/CI reporting and data-driven thresholds create attrition, confounding and analysis concerns. |
| Q17 | Tierney et al., 2025: longitudinal ACI Lysholm model | Moderate | High | Low | Low | Moderate | Moderate | High | Long follow-up attrition was substantial; mixed modeling and imputation were appropriate, but residual confounding and selected model reduction remain. |
| Q18 | Cook et al., 2025: OCA failure comparisons | Moderate | High | Low | Low | High | High | High | The source denominator/attrition was incompletely reported and factor estimates were unadjusted despite correlated patient and procedure characteristics. |
| Q19 | Yazdi et al., 2025: OCA threshold-outcome models | Moderate | High | Low | Low | Moderate | High | High | A ≥5-year responder cohort and many outcome-specific candidate-variable models create attrition/multiplicity and model-stability concerns. |
| Q20 | Wang et al., 2025: OCA failure model | Moderate | Moderate | Low | Low | Moderate | Low | Moderate | Large registry and interpretable adjusted estimates, but retrospective participation/follow-up and residual confounding remain. |
| Q21 | Ebert et al., 2025: 10-year MACI MRI models | Moderate | Moderate | Low | Low | Moderate | Low | Moderate | Long-term MRI cohort and reported CIs support inference, although selection into 10-year imaging and residual confounding remain possible. |
| Q22 | Weishorn et al., 2024: long-term MACI KOOS model | Moderate | High | Low | Low | Moderate | High | High | Only 59/150 treated patients entered the multivariable model; postoperative MOCART was included with baseline factors and CIs were not reported. |
| Q23 | Sato et al., 2024: preoperative WORMS correlations | High | High | Low | Low | High | High | High | Small selected mixed-procedure sample, no baseline PROM, unadjusted multiple correlations and incomplete control of key confounders. |
| Q24 | Bumberger et al., 2023: bipolar-lesion matched analysis | Moderate | High | Low | Low | Moderate | Moderate | High | Propensity matching addressed measured baseline factors, but procedure heterogeneity and marked follow-up attrition limit causal/prognostic interpretation. |
| Q25 | Byrne et al., 2023: claims-based OCA salvage model | Moderate | Low | Moderate | Moderate | Moderate | Low | Moderate | Large continuously enrolled claims cohort with adjusted estimates, offset by coding-based factor/outcome measurement and absent lesion detail. |
| Q26 | Kim et al., 2020: MSC survival Cox model | Moderate | Moderate | Low | Low | Moderate | Low | Moderate | Large long-term cohort with Cox estimates; single-center retrospective selection, overlapping prior cohort and residual treatment-selection confounding remain. |
| Q27 | Merkely et al., 2021: procedure-specific graft-failure analyses | Moderate | Moderate | Low | Low | Moderate | High | High | ACI model was adjusted but follow-up completeness was unclear and the published female-sex coefficient/OR are internally inconsistent; OCA results were unadjusted. |
| Q28 | Wright-Chisem et al., 2021: drug-allergy MCID models | Moderate | Moderate | Moderate | Low | Moderate | Low | Moderate | Predictor was self-reported and cohort overlap is probable, but models adjusted core demographic factors and retained null results. |
| Q29 | Gillinov et al., 2022: claims-based ACI revision model | Moderate | Low | Moderate | Moderate | Moderate | Low | Moderate | Continuous claims follow-up and adjusted estimates are strengths; diagnosis/procedure coding and unavailable lesion/baseline outcome data limit confounding control. |
| Q30 | Owusu-Akyaw et al., 2021: SF-36/defect-size IKDC-MCID model | Moderate | High | Low | Low | Moderate | Moderate | High | Only 91/141 patients had complete data and event-per-variable/model selection may be unstable despite reported ORs and CIs. |
| Q31 | Ramkumar et al., 2021a: OCA mental-health machine learning | Moderate | Moderate | Low | Low | Moderate | High | High | Feature rankings lacked factor-specific effects/CIs, many algorithms were compared, and cohort overlap precludes independent counting. |
| Q32 | Ramkumar et al., 2021b: OCA imaging machine learning | Moderate | Moderate | Low | Low | Moderate | High | High | Same 185-patient cohort as Q31; feature rankings lacked factor-specific effects/CIs and imaging-model multiplicity limits inferential interpretation. |
| QUIPS, Quality In Prognosis Studies; D1, study participation; D2, study attrition; D3, prognostic factor measurement; D4, outcome measurement; D5, study confounding; D6, statistical analysis and reporting. Ratings were low, moderate, or high risk of bias. Overall judgments used the highest domain rating and were not calculated as numerical scores. Supporting rationales are based on information reported in the primary publications. Estimates derived from the same analysis/model share an assessment ID and rationale. Q13 and Q14 represent the confirmed overlapping Kreuz cohort and were not counted as independent evidence. | | | | | | | | | |
